# Assessing the probability of introduction and spread of avian influenza (AI) virus in commercial Australian poultry operations using an expert opinion elicitation

**DOI:** 10.1371/journal.pone.0193730

**Published:** 2018-03-01

**Authors:** Mini Singh, Jenny-Ann Toribio, Angela Bullanday Scott, Peter Groves, Belinda Barnes, Kathryn Glass, Barbara Moloney, Amanda Black, Marta Hernandez-Jover

**Affiliations:** 1 The University of Sydney, Sydney School of Veterinary Science, Faculty of Science, Sydney, NSW, Australia; 2 Quantitative Sciences, Department of Agriculture and Water Resources, Canberra, ACT, Australia; 3 Research School of Population Health, Australian National University, Canberra, ACT, Australia; 4 NSW Department of Primary Industries, Orange, NSW, Australia; 5 NSW Department of Primary Industries, Menangle, NSW, Australia; 6 Graham Centre for Agricultural Innovation (An alliance between Charles Sturt University and NSW Department of Primary Industries), Charles Sturt University, School of Animal and Veterinary Sciences, Locked Bag 588, Wagga Wagga, NSW, Australia; 7 School of Animal and Veterinary Sciences, Charles Sturt University, School of Animal and Veterinary Sciences, Locked Bag 588, Wagga Wagga, NSW, Australia; Hokkaido University Graduate School of Medicine, JAPAN

## Abstract

The objective of this study was to elicit experts’ opinions and gather estimates on the perceived probability of introduction and spread of avian influenza (AI) virus in the Australian broiler and layer industry. Using a modified Delphi method and a 4-step elicitation process, 11 experts were asked to give initial individual estimates for the various pathways and practices in the presented scenarios using a questionnaire. Following this, a workshop was conducted to present group averages of estimates and discussion was facilitated to obtain final individual estimates. For each question, estimates for all experts were combined using a discrete distribution, with weights allocated representing the level of expertise. Indirect contact with wild birds either via a contaminated water source or fomites was considered the most likely pathway of introduction of low pathogenic avian influenza (LPAI) on poultry farms. Presence of a water body near the poultry farm was considered a potential pathway for introduction only when the operation type was free range and the water body was within 500m distance from the shed. The probability that LPAI will mutate to highly pathogenic avian influenza (HPAI) was considered to be higher in layer farms. Shared personnel, equipment and aerosol dispersion were the most likely pathways of shed to shed spread of the virus. For LPAI and HPAI spread from farm to farm, shared pick-up trucks for broiler and shared egg trays and egg pallets for layer farms were considered the most likely pathways. Findings from this study provide an insight on most influential practices on the introduction and spread of AI virus among commercial poultry farms in Australia, as elicited from opinions of experts. These findings will be used to support parameterization of a modelling study assessing the risk of AI introduction and spread among commercial poultry farms in Australia.

## Introduction

Although the primary source of avian influenza (AI) virus introduction into commercial poultry is believed to be from aquatic wild bird reservoirs [1–4], the mechanisms of its introduction and particularly subsequent spread have not been fully elucidated. A number of pathways of virus introduction, establishment and then spread have been proposed. Movement of humans (visitors, contractors, service personnel and farm personnel), vectors (wild birds, rodents, and insects), contaminated environment (air, water and dust) and other fomites (e.g., delivery trucks, clothing, and farm equipment) have all been hypothesized as potential spread pathways [5–10].

Australia has had seven highly pathogenic avian influenza (HPAI) outbreaks on chicken farms during the last 32 years, four of which have occurred in the last 10 years, suggesting that the frequency of outbreaks is increasing. In these past seven HPAI outbreaks in Australia, involving a total of 12 farms, all viruses were of subtype H7 and of Australian lineages but the definite mechanisms for spread between farms were not identified. Both H5 and H7 low pathogenic avian influenza (LPAI) viruses, which have the potential to mutate into HPAI, are endemic in wild birds of Australia [11]. It is believed that AI virus outbreaks in Australia occur via endemic LPAI exposure following contact with wild birds and then mutation to HPAI on poultry farms.

A resurgence of AI virus has been seen around the world in recent years. H5N1 occurred in several parts of Africa in 2014, with Nigerian farms and flocks particularly affected [12]. The emergence in January 2014 of a new highly pathogenic H5N8 AI virus in South Korea resulted in the destruction of more than 13 million birds in dozens of farms throughout the Korean peninsula. [13]. The origin of the 2014 outbreaks of the high pathogenicity H5N8 avian flu virus in Europe and in Japan can be traced to the Siberian summer breeding grounds of long-range migratory birds which provided a connection between different migratory flyways [14]. Forty seven million birds (7.5 million turkeys and 42.1 million egg-layer and pullet chickens) were killed in spring of 2015 in the USA either by or because of AI virus, with a cost to Federal taxpayers of over $950 million [15]. Forty of the one hundred and ten infected Minnesota flocks in the USA outbreak were introductions of HPAI virus directly from a wild waterfowl source. However, only two of the 77 cases in Iowa were introductions from wild birds, while virus spread between farms accounted for the remaining cases [15]. As of July 2017, HPAI outbreaks have been reported in Africa (Cameroon, Congo, Egypt, Niger, Nigeria, South Africa, Togo and Zimbabwe), USA, Asia and the Pacific (China, Chinese Taipei, India, Korea and Lao) and Europe (France, Germany, Italy, Montenegro, Netherlands, Russian Federation and Slovenia) with over 30 million poultry destroyed [16].

Although Australia is currently free from HPAI, the impacts of an epidemic on Australia’s commercial broiler and layer industries can be substantial [17]. New and emerging risks for the poultry industry in Australia need to be evaluated in the context of an increase in barn and free range farms. The Australian Egg Corporation Limited's annual report shows free range eggs now account for 50.6% of all grocery egg sales by volume, up from 20.3% per cent a decade ago [18]. Even though HPAI has not been isolated from wild birds in Australia, there have been a number of confirmed LPAI detections in wild birds in Australia [19].

In most outbreaks of AI in Australia, direct and indirect contact between wild waterfowl and poultry has been identified as an important introduction pathway for AI [20]. In at least two of the seven Australian outbreaks of HPAI, surface drinking water contaminated with waterfowl faeces was suspected to be the source of infection [21]. Poultry become infected after ingesting or inhaling the virus and can shed high concentrations of virus in respiratory secretions and faeces. In three of the seven outbreaks of HPAI in Australia there was suggestion of spread of virus from adjoining duck farms or an emu flock [22–24]. Fomites (personnel, animals, dust etc.) have been implicated as source of AI virus spread in at least two of the seven outbreaks in Australia [21, 23, 25]. Six of the seven outbreaks occurred in low poultry density regions and the potential for spread of HPAI between farms in higher density regions is not known [26].

Expert opinions are a valuable option for gathering knowledge in a field where accurate and unbiased field data are not available [27, 28]. Information on the probability of introduction and spread of AI among commercial poultry operations in Australia is not available. Routine sampling of Australian commercial chicken farms for LPAI is needed to generate data that can be used for accurate risk assessment. However, serological surveys are not considered an option in Australia. Information from countries in the Northern hemisphere is not transferrable to Australian conditions based on the differences in weather patterns, seasons, wild bird species, migratory bird patterns etc., which results in varied sources and pathways for introduction and spread of the virus in Australia. Moreover, all viruses identified in the previous HPAI outbreaks were of Australian lineages [19]. Data for avian influenza virus characteristics and behaviour especially in an Australian context is extremely scarce. To address this gap, the current study aimed to investigate the pathways of AI introduction and spread and the corresponding probabilities of these pathways, using an expert opinion exercise with a modified Delphi technique and a 4-step elicitation procedure [29] on different types of broiler and layer production operations. The results from this study will address gaps in knowledge and inform a scenario tree modelling study to estimate the AI risk of introduction and spread in the Australian poultry industry.

## Materials and methods

### Ethics statement

The study protocol, recruitment letter and participation information sheet (PIS) to be used in the expert elicitation exercises were approved by the University of Sydney Human Research Ethics Committee (Project Number: 2015/768).

### Selection of experts

Twelve poultry veterinarians and scientists were identified as experts, based on their experience in the Australian poultry industries, knowledge of the AI virus, knowledge of wild bird prevalence or involvement in the management of HPAI outbreaks in Australia or overseas. Each expert was invited to participate via an email letter that explained the aim and methods of the study and asked for a reply to provide consent to participate. On gaining consent, the questionnaire, participant information sheet (PIS) and instructions were emailed to participants who were asked to respond within two weeks. A reminder was sent to non-responders after two weeks.

### Study design

An expert elicitation exercise was conducted using a modified Delphi method. Experts were asked to complete an initial questionnaire, sent through e-mail, requesting probability estimates on various pathways and practices presented as scenarios. Following this, a face-to-face workshop was facilitated where individual and group averages of the initial estimates were presented and discussion between experts was encouraged. Experts were then invited to review and revise their individual answers using a final paper questionnaire.

### Questionnaire structure

The questionnaire consisted of five sections with questions arranged in a logical series of events and referring to: participant’s involvement in the poultry industry and knowledge of AI virus, probability estimates on: LPAI introduction into a shed (wild bird pathways and distance from water bodies), LPAI establishment in a shed, spread shed-to-shed on a farm (LPAI and HPAI), and spread farm-to-farm (LPAI and HPAI). A total of 41 questions were included: 3 related to participant experience, 29 probability elicitations, 4 open-ended questions (to list novel pathways not itemized in the questionnaire) and 5 calibration questions (responses to these questions provide basis for weighting experts’ opinions and infer the quality of the responses to the elicitation questions). The questionnaire is available as a supplementary information file **S1 Appendix**.

### Elicitation of probability estimates

Quantitative data were gathered using a 4-step elicitation procedure. Experts were asked to provide a four-point estimate that included the minimum, maximum and most likely values of the probability in the presented scenario and their level of confidence (between 50 to 100%) that the true value would fall within the interval created. The derived 80% intervals, representing the intervals with an 80% chance of including the true value, were calculated for each expert and for the group to be presented to participants at the workshop. This 4-step elicitation approach has been previously found to minimize participant overconfidence when compared to other methods for eliciting expert opinion [29].

According to Gigerenzer 2002 [30], greater accuracy of estimates is achieved when natural frequencies are requested compared to probabilities or percentages. Thus, a frequentist question format was used in the questionnaire, where for each question, the scenario referred to 100 typical operations of each of broiler barn (BB), broiler free range (BFR), layer cage (LC), layer barn (LB) and layer free range (LFR) types with a number of pathways (introduction or spread) listed in each scenario. Experts were asked the most likely, lowest and highest number of operations that would experience introduction or spread according to the different pathways considered, using hypothetical scenarios. Experts needed to elicit their responses for each of these pathways ranging from 0–100, and were provided with a guide on probabilities based on the Department of Agriculture and Water Resources terminology [31], as provided in Table 1.

**Table 1:** Probability categories used in the study (adapted from Biosecurity Australia 2001).

Qualitative Probability	Description	Natural frequencies The number of events out of 100
High	The event would be very likely to occur	70–100
Moderate	The event would occur with an even probability	30–70
Low	The event would be unlikely to occur	5–30
Very low	The event would be very unlikely to occur	0.1–5

### Face-to-face workshop

The workshop commenced with a review of the study aims and of the methodology used for expert opinion elicitation. Responses to all questions, including the most likely values and the derived 80% intervals on an individual basis as well as group averages were presented without identifying the experts. For each question, an initial dialogue with the participants allowed a clear and common linguistic interpretation of the question and agreed set of underlying assumptions. The participants were then prompted to discuss the topic, where the aim was not to reach a consensus but to capture the range of opinions and thus quantify the extent of uncertainty around the estimate. Time was allocated after discussion on each question to reassess and revise the initial estimates if desired. The final estimates were then collected while reassuring the participants that all individual responses would remain private and confidential.

### Data analysis

Initial response data was coded and entered into a database file (Excel™; Microsoft Corp., Redmond, WA) (**S1 Dataset**). The corresponding 80% intervals were obtained using a LogNormal transformation, as the experts’ estimates were not considered normally distributed [29]. The most likely values and the 80% interval for each participant were graphed for all 29 elicitation questions. Group means were also displayed for each probability. To calculate the final estimates for each expert the same approach was used. Final estimates of natural frequencies (the number of events out of 100) were collated, and the 80% derived intervals and graphical representations of the answers for each question were obtained as before.

To obtain a single estimate for each frequentist question, responses from all experts were considered and combined. Variability and uncertainty in each pathway probability were incorporated using probability distributions to represent experts’ estimates and a Monte-Carlo stochastic process [32]. Each expert response (most likely, lowest and highest number of operations) was modelled using a pert distribution. This distribution, which is frequently used to model expert opinion, is four times more sensitive to the most likely value than to the minimum and maximum values [32]. To combine responses of all experts for each question and incorporate the differences in experts’ opinion, a discrete distribution was used. This distribution considered the probability estimate from each expert, which was obtained with a pert distribution, and the weight given to each expert, according to their response on calibration questions. The output probability distribution of each combined estimate for each frequentist question was calculated using a Monte-Carlo stochastic simulation process with @Risk 6.0 (Palisade Corporation, USA). Each simulation comprised 10,000 iterations, sampled using the Latin hypercube method, with a fixed random seed of one. Results were graphed and the raw median and range of responses were determined.

#### Weighting of expert’s opinion

Experts’ opinions were weighted based on their response to calibration questions. These questions were classified as having a focus on the poultry industry or wild birds/AI virus. Experts’ expertise was ranked in three categories (low, medium and high) for each of these two areas. All elicitation questions were also categorized as either industry or wild bird/AI virus related, and weights allocated to responses from experts based on their ranking in that area of expertise with 1 being low and 3 being high (**S1 Dataset**).

## Results

### Participants for the expert opinion workshop

Eleven of the 12 selected experts (92%) completed the questionnaire, either electronically (10/12) or in hard copy and scanned (1/12). Ten of the 12 experts (83%) participated in the workshop, all of whom had completed the questionnaire. The responses of all 10 experts were analysed for each section of the questionnaire and used for the estimation of probabilities.

### Professional background of participants

Of the 10 participants, six were poultry veterinarians (three employed in private consultancy, two in poultry companies and one in the university sector) with an average of 35.8 years of experience in the poultry industry. The other four were: a virologist (7 years of experience in AI virology), an epidemiologist (30 years of experience), a wild bird expert (10 years of wild bird surveillance experience) and an avian diagnostic pathologist (33 years of experience).

### Calculated estimates

In a majority of cases (92.8%), the experts’ combined probability had narrow ranges, showing significant consensus in individual responses. In these instances where consensus was reached, the median of all combined probability distributions was used as the final estimate, with the ranges depicted as the 5 and 95 percentiles of the probability distribution. In a few cases (3.8%) participants’ combined probability distributions displayed wide ranges, probably due to the wide intervals placed around estimates by individual participants and the lower levels of confidence in their answers, however the estimates showed a unimodal distribution and a single median and range was applied. In the remaining cases (3.4%), participant’s estimates resulted in bimodal probability distributions. When plausible reasoning supported the different positions taken by the experts, both options were retained as alternative explanations and a single median estimate was not used.

### The probability of LPAI introduction into a shed

#### Wild bird pathways

Four pathways were presented to the experts under the wild bird pathways category: direct contact with the wild bird, indirect contact via a contaminated water source, indirect contact via fomites and indirect contact via aerial dispersion of faeces (dust, fans etc.). The definition of direct contact was clarified for experts as direct physical contact with a wild bird or its faeces for the purpose of this elicitation.

**Fig 1:**
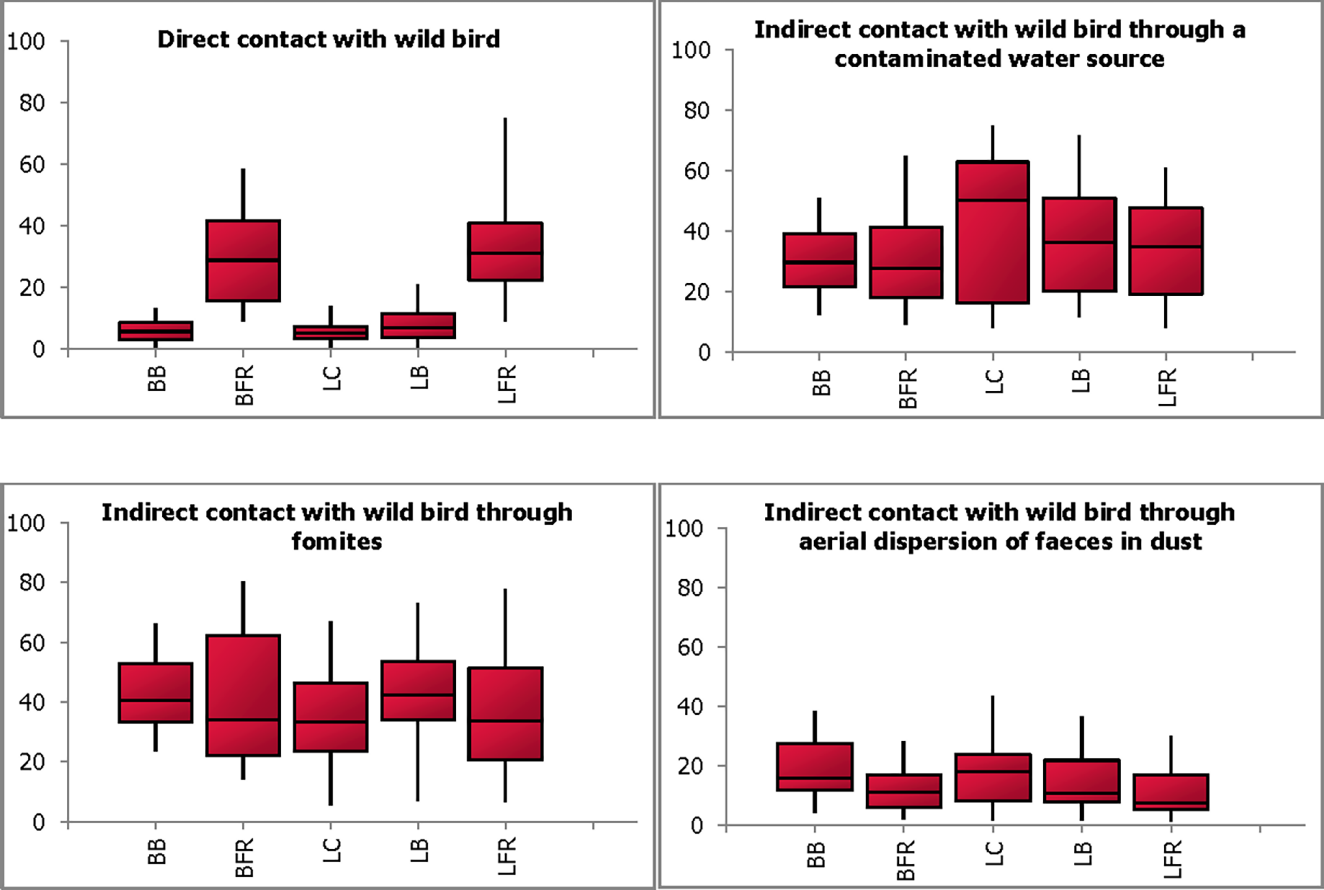
A box-and-whisker plot of expert’s combined probability distributions for introduction of avian influenza (AI) by different pathways. The plot shows the median, the lower and upper quartiles (25% and 75%) (enclosed by shaded boxes) and the lower (5%) and upper (95%) values as whiskers in each production type (broiler barn (BB), broiler free-range (BFR), layer cage (LC), layer barn (LB) and layer free-range (LFR)) for each of the pathways.

Fig 1 illustrates box-and-whisker plots of combined probability distributions for all five types of chicken operations: broiler barn (BB), broiler free range (BFR), layer cage (LC), layer barn (LB), and layer free range (LFR) in the four presented scenarios. The likelihood of introduction of LPAI through direct contact with wild bird was estimated to be very low for BB (5.7%; 0.8–13.2%), LC (5.1%; 0.4–14.0%) and LB (6.8%; 0.3–21.2%) as compared to BFR (28.9%; 9.1–58.7%) and LFR (31.0%; 8.9–75.1%). This was primarily due to a consensus in opinion that chickens while on range would have a greater probability of direct contact with a wild bird.

The most likely pathways of introduction of AI across all operation types were indirect contact to wild birds either via fomites (median ranging from 33.3% (5.6–67%) to 42.2% (6.9–73.1%)) or via a contaminated water source (median ranging from 29.7% (12.6–50.8%) to 50.1% (8.1–74.4)), while aerial dispersion of faeces was considered less likely to occur (median ranging from 7.2% (1.4–30.0%) to 17.8% (1.5–43.2%)). Although generally experts believed that water used on-farm is usually treated, two experts were of the opinion that water treatment was not adequate on all farms. Water treatment in broiler farms was considered to be of higher standard due to tighter regulations as well as better access to technical expertise, when compared to independently owned layer farms. Estimates for indirect contact through contaminated water and fomites resulted in wide intervals, indicating there were divergences in opinions and significant uncertainty around these estimates. In comparison, probability estimates for direct contact and indirect contact through aerial dispersion had narrower intervals indicating lower levels of uncertainty and greater consensus among experts.

#### Distance from unprotected water body

Water bodies on chicken farms may be dams that are used to provide drinking water, cooling, irrigation (of range areas), and containment of run-off, or natural water bodies such as lakes, rivers and irrigation channels.

**Fig 2:**
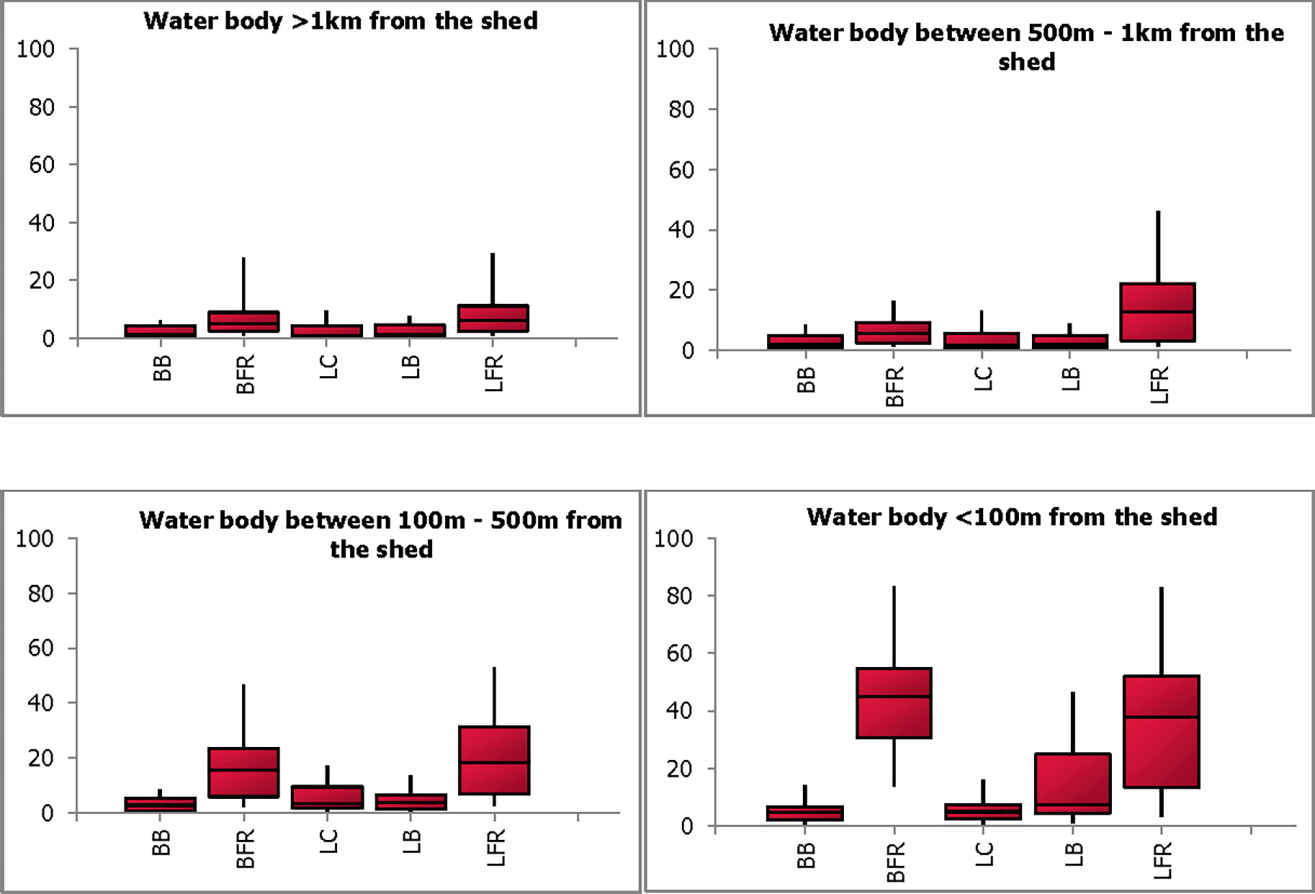
Combined probability estimates for introduction via water body at different distances from the shed. The plot shows the median, the lower and upper quartiles (25% and 75%) (enclosed by shaded boxes) and the lower (5%) and upper (95%) values as whiskers in each production type (broiler barn (BB), broiler free-range (BFR), layer cage (LC), layer barn (LB) and layer free-range (LFR)) for each of the distances.

Experts were presented with a scenario where the presence of an unprotected water body source resulted in the congregation of wild birds, with an assumption that 50% of the wild birds were infected with LPAI. The combined probability estimates from all experts for LPAI introduction, if there were 100 sheds located at each of the specified distances ranging from <100m to >1 km, from the water body, are presented in Fig 2. (Note: the question did not consider use of water body as a source of drinking water for chickens).

There was a close consensus in the probability estimates of introduction of AI virus at all specified distances, with very low estimates for all production types except free range (Fig 2). The estimates increased with decrease in distance between the shed and the water body (eg. median 1.2% (0–7.9%) to 7.4% (1.2–46.6) in LB systems). The estimates were highest for both broiler and layer free range operations at <100 m distance of shed from the water body (median 45%; 13.7–83.3% and 37.7%; 3.3–82.7% respectively).

Two modes of introduction of LPAI, related to the distance from the water body, were discussed at the workshop. The first was the movement of wild birds (duck, waterfowl etc.) onto the range area due to its proximity to the water body. Ducks were considered to be more prevalent on pastures. The other mode was the aerial transmission of virus from the contaminated grass area around the dam where the wild birds congregate.

### LPAI establishment in a shed

#### Detection of LPAI based on mild clinical signs

The probability of LPAI being detected on-farm (where infection had been established in the shed and birds were shedding the virus), assuming mild clinical signs, such as mortality/morbidity/decrease in production etc. are present during one week, was estimated to be very high across all the different production types (median ranging from 79.6% to 90.7%). However, the estimates were higher and confidence intervals smaller for both BB (90.4%; 75.0–97.2%) and BFR (90.7; 61.9–97.2%) operations, mainly due to the opinion that mild clinical signs due to LPAI would be most likely detected in broilers within one week due to daily monitoring and inspection of birds. In comparison, any change in production measures for layers would only be evident if the production losses were significant over a few weeks.

#### Mutation of LPAI to HPAI

**Fig 3:**
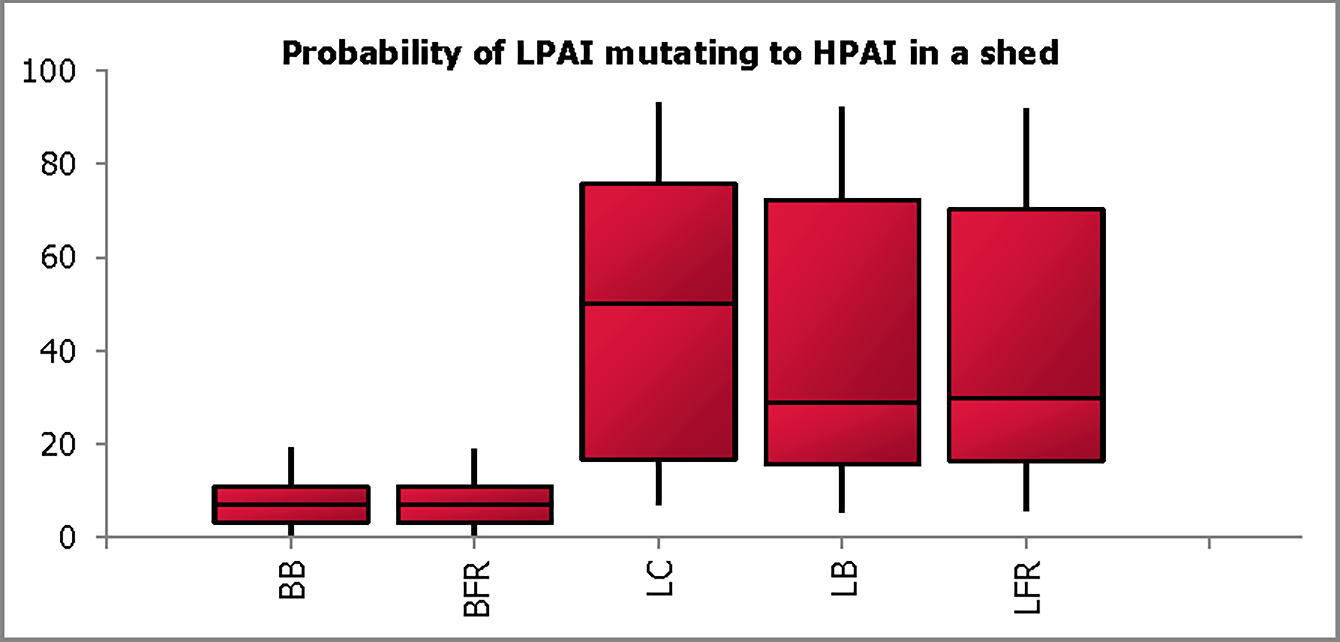
Combined probability estimates for probability of LPAI mutating to HPAI in a shed. The plot shows the median, the lower and upper quartiles (25% and 75%) (enclosed by shaded boxes) and the lower (5%) and upper (95%) values as whiskers in each production type (broiler barn (BB), broiler free-range (BFR), layer cage (LC), layer barn (LB) and layer free-range (LFR)).

Current information on the mutation of H5 and H7 subtypes of LPAI in infected chickens to their highly pathogenic phenotype is limited. Experts’ estimates on the probability of H5 and H7 LPAI mutation to HPAI, after infection had been established in the shed, are shown for all production types in Fig 3. The estimates were low for broilers (6.9%; 0.0–19.2%) as one production cycle of 45–49 days was not considered long enough by experts for the mutation to effectively occur.

In contrast, the estimates for layers were higher (49.9%; 7.0–93.1%, 28.9%; 5.5–92.3%, and 29.7%; 5.7–92.0 for LC, LB, and LFR respectively); however, the estimated 5 to 95% probability intervals were very wide. The main factor discussed contributing to these higher estimates was their long production cycle (75–90 weeks), during which layers could remain exposed to virus for a longer duration (number of possible bird to bird transmission within flock life time), allowing for a higher chance of mutation to occur. Density and proximity of birds to each other were also considered important factors, which are seen with higher probability estimate obtained for caged layer. Other factors considered were the lack of restriction in bird-to-bird contacts on site and the possibility of early intervention to LPAI infection reducing the incidence of mutation.

### Probability of spread from shed-to-shed on a farm

The median probability estimate for spread of LPAI infection to at least one other shed on the property was significantly different for broiler and layer operations, with lower estimates for both BB (36.9%; 7.7–78.9%) and BFR (45.2%; 14.3–75.41%) compared to LC (70.8%; 9.0–95.0%), LB (72.8%; 12.3–95.7%) and LFR (77.9%; 23.0–96.0%). However, the estimated intervals were wide for individual experts as well as the combined interval in all operations, showing a low level of expert confidence in their estimates.

**Fig 4:**
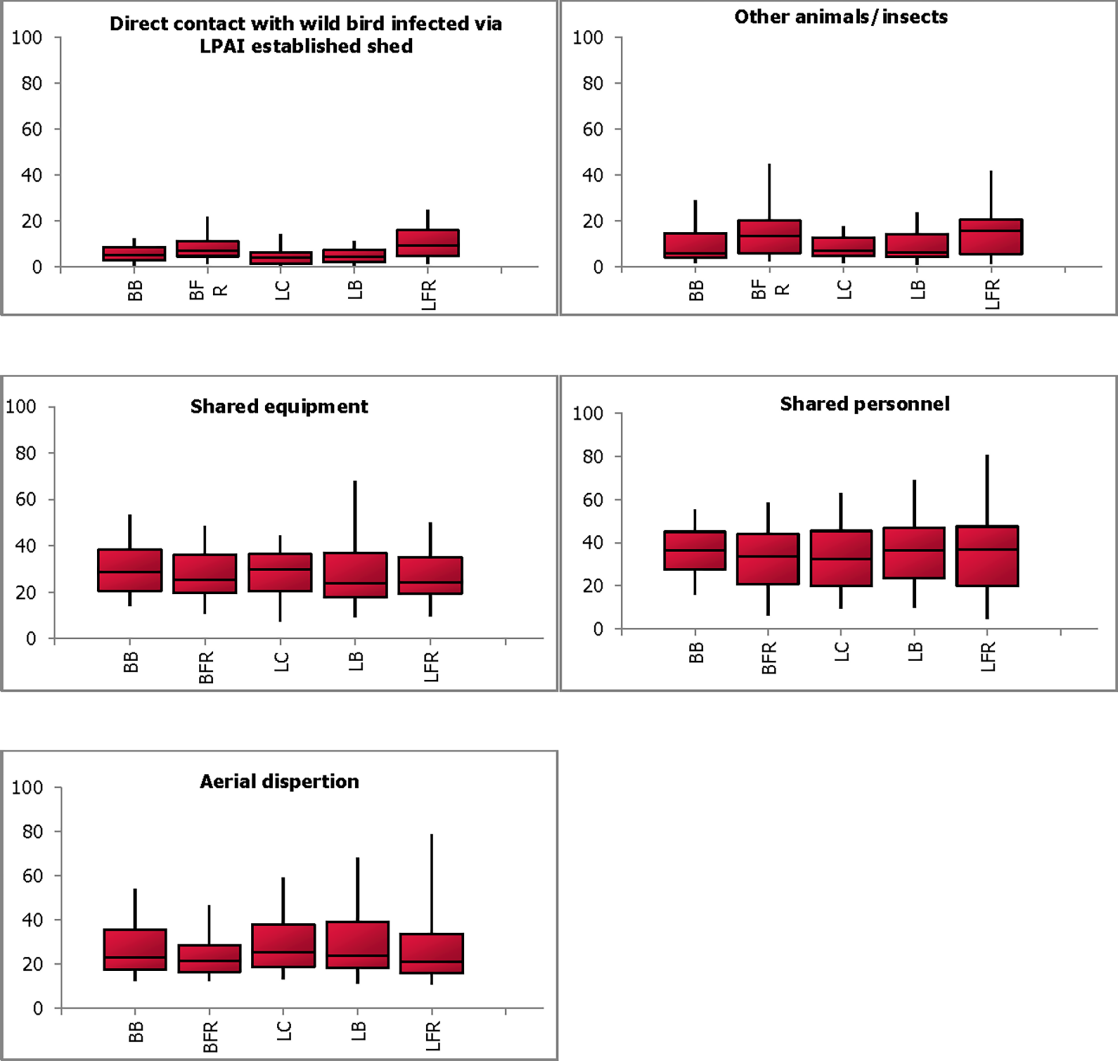
Combined probability estimates for spread of LPAI infection to at least one other shed on the property via different pathways. The plot shows the median, the lower and upper quartiles (25% and 75%) (enclosed by shaded boxes) and the lower (5%) and upper (95%) values as whiskers in each production type (broiler barn (BB), broiler free-range (BFR), layer cage (LC), layer barn (LB) and layer free-range (LFR)) for each of the pathways.

For spread of LPAI from a shed where infection had already been established to other sheds on the property, probability estimates for suggested pathways are shown in Fig 4 (expert’s estimates for all pathways needed to add to 100 to allocate probabilities for each pathway). Shared personnel (median ranging from 32.5%; 9.6–63.2% to 36.7%; 16.0–55.4%), followed by shared equipment between sheds (23.7%; 9.3–67.8% to 29.7%; 7.3–44.3%) and aerial dispersion of virus (21.1%; 10.9–78.9% to 25.4%; 13.0–59.0%) were implicated as the most likely routes of spread of LPAI infection between sheds. Direct contact with wild birds infected from a LPAI established shed (4.1%; 0.5–11.4% to 9.4%; 1.6–24.9%) was not considered as a likely pathway, while spread via other insects/animals was considered to pose a higher risk on free range farms (13.3%; 2.2–44.7% for BFR and 15.6%; 1.1–41.8% for LFR) than enclosed farms for both broilers and layers (Fig 4).

The likelihood of spread of HPAI from shed to shed on a farm was high for all operation types across broilers and layers (median ranging from 71.7%; 42.5–98.7% to 79.2%; 53.2–98.5%). The general consensus was that HPAI would invariably spread from shed to shed on an infected property and this was validated by the history of earlier outbreaks of AI in Australia. One expert however pointed out that since HPAI was easy to detect as compared to LPAI, interventions would be implemented more quickly reducing the likelihood of spread to another shed. However, this was challenged by other experts who stated that the higher shedding of virus by HPAI infected chickens would invariably increase the probability of spread on a farm.

### Probability of spread from farm-to-farm

Probability estimates of spread of LPAI infection to at least one other chicken farm of any operation type before it is detected or intervened was considered to be low (median between 7.7; 0.0–26.5% to 13.6%;1.7–48.4%) and lowest for broiler farms. In contrast, HPAI spread was considered to be more likely in all operation types (Table 2). However, the confidence intervals around these estimates were very large. The experts indicated that the probability of spread of HPAI from one farm to other farms depended on various factors. While some experts believed that HPAI detection and response would occur in a shorter time period than for LPAI, reducing the time for the potential spread, others believed that a high level of spread of HPAI could occur due to higher shedding of the virus in a short period of time compared to the level of shedding in LPAI outbreaks. In addition, one of the experts also indicated that in past outbreaks, HPAI spread occurred due to poor biosecurity practices and not due to a lack of detection. Opinion was divided on the spread of virus between broiler farms as compared to layers, with some experts believing that spread in broilers was less likely than in layers due to tighter biosecurity between farms, whilst others considering that the higher transport and personnel movement among broiler farms would likely cause the spread of the virus.

**Table 2:** Combined probability estimates (Median (5% and 95% estimate interval)) for spread of LPAI and HPAI infection from one farm to another.

	Broiler barn	Broiler free range	Layer cage	Layer barn	Layer Free range
**LPAI**	7.70 (0.0–26.5)	8.1 (0.0–34.7)	13.4 (1.7–45.5)	13.6 (1.8–48.4)	13.5 (1.7–48.8)
**HPAI**	23.0 (5.6–82.5	23.0 (7.0–83.1)	23.6 (6.5–61.4)	24.1 (6.5–78.9)	25.9 (13.9–75.3)

**Fig 5:**
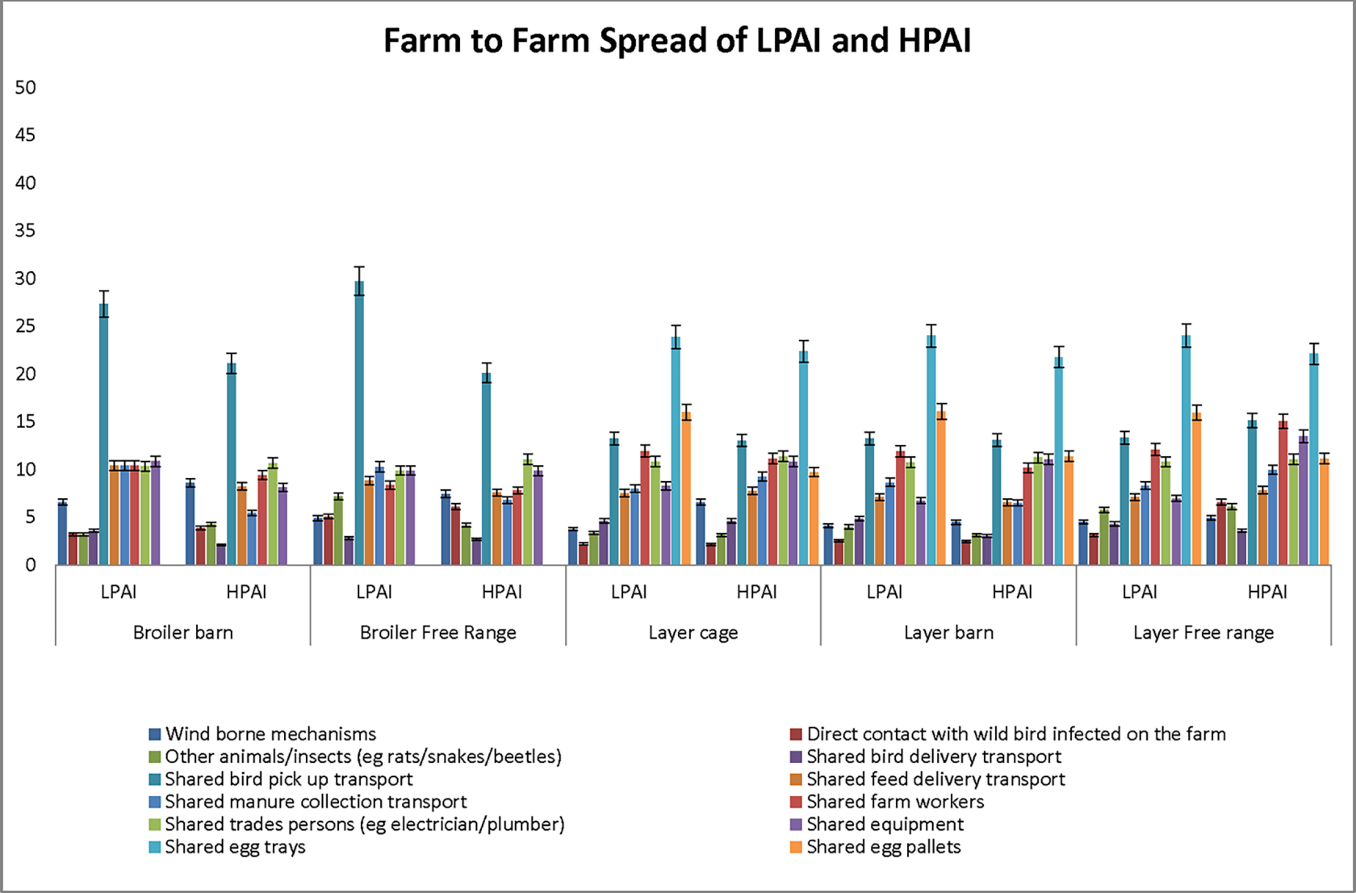
Combined probability estimates (median % ± 5% error) for spread of LPAI and HPAI infection to at least one other farm via different pathways.

Twelve similar hypothetical pathways for spread of LPAI and HPAI between farms were presented to participants, ten that applied to broiler farms and twelve to layer farms. Of the various pathways, shared bird pick up transport was considered to be most likely pathway of spread from broiler farms for both LPAI (27.3%; 11.6–61.1% for BB and 29.7%; 12.0–63.6% for BFR) and HPAI (21.1%; 4.1–46.8% for BB and 20.2%; 3.3–48.1% for BFR), while shared egg trays (24%; 4.3–42.8% each for LC, LB and LFR for LPAI and 22.4%; 8.0–47.4% for LC, 21.8%; 8.1–49.9% for LB and 22.1%; 8.0–54.3% for LFR for HPAI) and pallets (16%; 4.32–36.4% each for LC, LB and LFR for LPAI and 9.7%; 4.4–41.7% for LC, 11.4%; 4.1–50.0% for LB and 11.1%; 4.4–48.3% for LFR for HPAI) were considered to be most likely pathways for the layer operations (Fig 5).

Pick up of birds during the thinning-out process (partial depopulation at various intervals to satisfy market demand for birds at different weights), and pick-up of spent hens and restocking of pullets on layer farms, were considered while estimating the probability of spread via pick-up trucks. In addition, pick up of dead birds from a farm was also considered. Dead or partially dead bird (inadequate methods of mass culling could result in partially dead birds) pickups were considered a substantial threat to spread of LPAI and HPAI on broiler farms. For most broiler farms, dead birds are frozen, and collected at a later date. For layers, the number of dead birds is small and does not justify pick up. These birds are either disposed of on-site or moved off-site by the farmer.

For layer operations, the use of shared egg trays and pallets were identified as the most likely pathway of spread of LPAI from farm to farm. Even though larger farms have their own grading floors, there is risk of transmission from smaller farms that send their eggs to larger farms for grading. In addition, it was suspected that the trucks carrying eggs were not disinfected between farms and would travel long distances, thus spreading the infection farther from the farm of origin. Shared bird pick up transport (15.1%; 4.9–44.0%), shared farm workers (15.1%; 3.4–49.4%), and shared equipment (13.5%; 1.0–37.2%) were other pathways that were considered to be of risk for HPAI in LFR (Fig 5).

Opinion was also divided over the manure truck being a source of spread. One expert suggested that since manure pickup occurred after the birds had been removed, it would not be effective in spreading the virus, while other experts were of the view that the lack of clean-up of these trucks between manure pickup and litter delivery would contribute to the likely spread. Moreover, manure trucks going past other poultry farms also posed threat of spread via wind mediated pathways.

Although wind-borne mechanisms were considered less likely to cause spread of LPAI and HPAI to other farms (median ranging from 3.8%; 0.0–31.5% to 8.61%; 0.0–69.2%) for all poultry operations, it was believed that long distance aerosol transmission was not possible. One expert was of the opinion that spread was dependent on the proximity of the neighbouring farms and that aerosol transmission could be a possibility in areas where there were clusters of farms.

Other animal /insects were also considered as potential pathways, with rats and snakes being identified as mechanical vectors that could spread the infection between farms. There is a possibility of these animals acting as vectors by transporting contaminated material between locations and either directly infecting poultry or contaminating feed, especially when they have access to poultry houses and storage rooms on farms. Direct contact between high virus shedding chickens and wild bird, especially on free range farm types, was also considered a possible pathway of infecting wild birds and hence chickens on another farm.

One of the pathways that was not included in the questionnaire but was mentioned by a number of experts for spread of LPAI and HPAI was the movement of company service people between farms. Experts indicated that servicemen visited several farms within a company in a day and that the biosecurity measures were not followed optimally. Vaccination crews were also suggested to be a potential pathway of spread.

Other novel pathways of spread suggested by experts included: farm stays, raptors and predators moving dead or infected chickens from one farm to the other, especially those that could carry but not be infected themselves. A number of bridge avian and non-avian species (e.g. cats, sparrows, hawks, eagles etc.) were implicated as spread pathways.

## Discussion and conclusion

A modified Delphi method [29] was used to elicit experts’ opinion on the potential pathways of introduction and spread of AI virus among different poultry enterprises in Australia. The estimates were able to distinguish the level of risk perceived by the experts in cage versus barn versus free range production systems for both broiler and layer farms. Ten experts completed the elicitation process including completion of an electronic questionnaire, participation in a face-to-face workshop and revision of initial estimates. Advantages of this approach over the traditional Delphi method were that participants were allowed to share knowledge in a semi-structured group discussion, and potential psychological and social sources of bias due to group interaction were limited because experts answered questionnaires anonymously during the workshop. The 4-step interval elicitation procedure used in this method has been reported to reduce overconfidence [29]. A significant consensus was reached for 92.7% of the frequentist questions, while the rest either showed wide intervals indicating significant level of uncertainty due to low levels of confidence or bimodal frequencies indicating disagreement between groups of experts displaying the significant gaps of information in this field. The probability estimate intervals obtained in this study will be used as input parameters in scenario trees for risk analysis of introduction and spread of AI virus on commercial Australian chicken meat and egg farms.

Of the introduction pathways, the most likely in all types of poultry farms was indirect contact with wild birds, either via fomites, or via a contaminated water source. In several historical epidemics of HPAI, initial introduction of H7 LPAI viruses into a poultry flock has been speculated to be from wild waterfowl that contaminated surface water which was used for drinking [25, 33–35]. Indirect contact via fomites (equipment, vehicles or people contaminated with virus) has also been previously implicated for introduction of AI [33, 36, 37]. Direct contact with a wild bird or its faeces was considered to be more likely on free range broiler and layer enterprises as compared to the indoor housing systems. Surveillance activities from 2005 to 2008 in Australia reported circulation of LPAI virus subtypes in wild birds, with LPAI detection rates of 0.51% in shorebirds and 2.4% in waterfowl [38]. A recent study [19] also showed dominant and widespread prevalence of LPAI H5 subtypes in a surveillance study from 2008–2012. In the seven HPAI out-breaks reported in poultry in Australia, introduction of LPAI viruses from wild birds and subsequent mutation has been hypothesized as the most likely origin of these outbreaks [19, 38, 39].

Proximity to water bodies has been implicated as a risk for introduction of AI virus in a number of epidemiology studies, and water bodies considered repositories for AI virus with the water an important vehicle for AI virus transmission between migratory birds and poultry [35, 40–47]. It is however not clear if the distance from the water body has any effect on the probability of introduction of AI virus. Presence of a water body near the poultry farm was considered a potential pathway for introduction in this study, primarily if the farm was free range and if the water body was within 500 meters from the shed. Two features considered relevant to these estimates were the presence of green pastures on broiler free range farms, thus attracting ducks and waterfowl, and aerial dispersion of virus from contaminated grass around water bodies to the dust in and around the shed. Influenza viruses can remain infectious for many days in poultry litter and virus-contaminated droppings have been reported to serve as a source of infectious material, via dispersal into the environment through dust, for susceptible birds [7, 10, 48].

Experts generally provided high probability estimates for LPAI detection on all type of farms, however, detection on broiler farms (both BB and BFR) was considered more likely than on layer farms, when morbidity and mortality was evident in the shed, indicating frequent monitoring and inspection of birds on broiler farms as compared to layers (where a change in production parameters would take weeks to be detected). Although production parameters do not usually change in LPAI-infected flocks as dramatically as they do in HPAI, increased mortality, decreased egg production, and decreased feed and water consumption are common in LPAI virus-infected flocks [49–56]. However, even though the expert’s estimates for detection via these signs were high for all production types, morbidity and mortality was never reported as indicators in any LPAI H5/H7 outbreaks in Australia. LPAI viruses are often shed from clinically normal chickens and birds showing minimal clinical signs. Further, LPAI viruses tend to cause disease when chickens are co-infected with other pathogens or are subject to environmental stresses [57] making it even more difficult to associate the mild clinical signs with AI virus. In addition, the pattern of signs also varies with the flock. Nevertheless, some flocks infected with LPAI viruses would be detected only by routine testing.

Early detection of LPAI outbreaks can reduce the likelihood of mutations and thus large epidemics [58]. In this study, mutation of H5 and H7 LPAI to HPAI was considered to be highly unlikely in broilers due to the shorter length of their production cycle making it less conducive for an effective mutation to establish in that time. However, from previous Australian outbreaks it can be noted that a relatively rapid acquisition of virulence occurred (3–4 weeks) once the viruses entered the chicken flocks. However, even though the estimates for the probability of this mutation occurring on layer farms was relatively high, the wide intervals around the estimates showed that experts were not confident if a longer production cycle could be attributed as a reason for establishment of the mutation and there was a gap in knowledge about the rate and frequency of the mutation. Moreover, LPAI had not been identified in poultry in any of the past HPAI outbreaks in Australia. One of the suggested reasons for this has been that the samples obtained from farms were cultured, which meant that only the dominant viruses in the pool could be detected. Next generation sequencing may be a better methodology for detection of LPAI, especially when samples were quite compromised and thus show low viral loads [59].

Spread of LPAI from shed to shed on a farm was mainly attributed to shared personnel and equipment, although fomites and wild birds infected from an affected shed were also considered as relevant pathways in free range broiler and layer farms. Once an AI virus has entered a poultry flock, it can spread within the farm by both the faecal–oral route and aerosols, due to the close proximity of the birds [5, 6, 10, 60, 61]. A high likelihood of spread from shed to shed was anticipated for HPAI, due to high levels of virus shedding, even though it was believed that HPAI would be detected much earlier than LPAI. Though particular HPAI subtypes, such as H5N1, are expected to be detected relatively rapidly, with some analyses indicating no longer than one week [62], evidence from experimental work using the H7N7 subtype implicated in an outbreak in the Netherlands in 2003, suggested that HPAI may have been left to circulate within a flock undetected for 11–15 days [63].

Shared pick up transport was considered to be the most likely pathway of spread between broiler farms for both LPAI and HPAI. Transportation has been implicated as a potential pathway in a number of earlier outbreaks [6, 7, 19, 64, 65]. Timely and effective disposal of dead birds was an issue during the latest outbreak in USA in 2015, where depopulation of farms could not happen fast enough and transportation of infected dead birds could have been a contributing factor to the level of spread seen [15].

In the case of layer farms, shared egg trays and pallets accounted for the most likely pathway of spread between farms. During the Netherlands epidemic in 2003, increased risk was implicated in the form of high numbers of contacts between farms via cardboard egg trays used for removal of eggs during the epidemic [66]. Egg trays were also implicated as a spread pathway in spatial analysis of LPAI H5N2 outbreaks in Japan in 2005 [67] and as a risk factor for spread of HPAI H5N1 in commercial poultry farms in Kano, Nigeria [68].

This expert opinion exercise was meant to address knowledge gaps in introduction and spread pathways for AI in the Australian context. The probability estimates provided by experts were very varied in some cases and need to be interpreted with caution. While the biases of group discussion were removed by discussing the assumptions for each question, there is still a level of subjectivity in the estimates provided. The uncertainty and lower level of confidence for questions such as probability of mutation and probability of virus detection may have stemmed from the lack of mention of specific H5 and H7 strains in the question and also from the low number of AI occurrences to date in the Australian poultry sector. Also in hind sight, the questionnaire could have been better designed to avoid repetitive questions for each poultry sector separately, thereby reducing the overall length of the form. Nevertheless, the expert elicitation exercise provided useful information for input parameters in a risk assessment of the introduction and spread pathways.

The identified pathways of importance emphasise biosecurity and suggest a need for strict regulation of movements on farm, waterfowl deterrents and water treatment requirements. Lack of biosecurity practices addressing shared personnel and equipment were associated with spread of infection on farms and needs to be improved by either disinfection between sheds or allocation of dedicated personnel and equipment per shed. Biosecurity also needs to be strictly adhered to by shared transport drivers to ensure proper disinfection between farms and by avoiding multiple farm pickups. Cardboard egg trays and pallet movements need to be restricted for layer farms with better uptake of plastic or colour coded trays and adherence to single farm egg handling on grading facilities at any given time.

In conclusion, this elicitation of expert’s opinion provided useful information to address the pathways considered important in the introduction and spread of avian influenza. The various estimates generated in this paper will be used as input values in a risk assessment to inform on-farm biosecurity practices to mitigate risk, and also help in validating some of those estimates.

## Supporting information

S1 Appendix A copy of the questionnaire. (DOCX)

S1 Dataset Minimal dataset. (XLSX)

## References

[1] Webster RG, Bean WJ, Gorman OT, Chambers TM, Kawaoka Y. Evolution and ecology of influenza A viruses. Microbiological reviews. 1992;56(1):152–79. ; PubMed Central PMCID: PMC372859. 1579108 10.1128/mr.56.1.152-179.1992 PMC372859

[2] Alexander DJ. A review of avian influenza in different bird species. Veterinary microbiology. 2000;74(1–2):3–13. . 10799774 10.1016/s0378-1135(00)00160-7

[3] de Jong MC, Stegeman A, van der Goot J, Koch G. Intra- and interspecies transmission of H7N7 highly pathogenic avian influenza virus during the avian influenza epidemic in The Netherlands in 2003. Revue scientifique et technique. 2009;28(1):333–40. . 19618636 10.20506/rst.28.1.1859

[4] Alexander DJ. An overview of the epidemiology of avian influenza. Vaccine. 2007;25(30):5637–44. doi: 10.1016/j.vaccine.2006.10.051 17126960 10.1016/j.vaccine.2006.10.051

[5] Fusaro A, Tassoni L, Milani A, Hughes J, Salviato A, Murcia PR, et al Unexpected Inter-farm Transmission Dynamics During a Highly Pathogenic Avian Influenza Epidemic. Journal of virology. 2016 doi: 10.1128/JVI.00538-16 . 27147741 10.1128/JVI.00538-16 PMC4936132

[6] Hernandez-Jover M, Schemann K, East IJ, Toribio JA. Evaluating the risk of avian influenza introduction and spread among poultry exhibition flocks in Australia. Preventive veterinary medicine. 2015;118(1):128–41. doi: 10.1016/j.prevetmed.2014.11.018 . 25496909 10.1016/j.prevetmed.2014.11.018

[7] Ssematimba A, Hagenaars TJ, de Wit JJ, Ruiterkamp F, Fabri TH, Stegeman JA, et al Avian influenza transmission risks: analysis of biosecurity measures and contact structure in Dutch poultry farming. Preventive veterinary medicine. 2013;109(1–2):106–15. doi: 10.1016/j.prevetmed.2012.09.001 . 22998848 10.1016/j.prevetmed.2012.09.001

[8] Poolkhet C, Chairatanayuth P, Thongratsakul S, Kasemsuwan S, Rukkwamsuk T. Social network analysis used to assess the relationship between the spread of avian influenza and movement patterns of backyard chickens in Ratchaburi, Thailand. Res Vet Sci. 2013;95(1):82–6. doi: 10.1016/j.rvsc.2013.02.016 . 23528640 10.1016/j.rvsc.2013.02.016

[9] Haase M, Starick E, Fereidouni S, Strebelow G, Grund C, Seeland A, et al Possible sources and spreading routes of highly pathogenic avian influenza virus subtype H5N1 infections in poultry and wild birds in Central Europe in 2007 inferred through likelihood analyses. Infection, genetics and evolution: journal of molecular epidemiology and evolutionary genetics in infectious diseases. 2010;10(7):1075–84. doi: 10.1016/j.meegid.2010.07.005 . 20624487 10.1016/j.meegid.2010.07.005

[10] Vieira AR, Hofacre CL, Smith JA, Cole D. Human contacts and potential pathways of disease introduction on Georgia poultry farms. Avian diseases. 2009;53(1):55–62. doi: 10.1637/8364-051608-Reg.1 . 19432004 10.1637/8364-051608-Reg.1

[11] Haynes L, Arzey E, Bell C, Buchanan N, Burgess G, Cronan V, et al Australian surveillance for avian influenza viruses in wild birds between July 2005 and June 2007. Australian veterinary journal. 2009;87(7):266–72. doi: 10.1111/j.1751-0813.2009.00446.x . 19573149 10.1111/j.1751-0813.2009.00446.x

[12] Adegboye OA, Kotze D. Epidemiological analysis of spatially misaligned data: a case of highly pathogenic avian influenza virus outbreak in Nigeria. Epidemiology and infection. 2014;142(5):940–9. doi: 10.1017/S0950268813002136 . 24001574 10.1017/S0950268813002136 PMC9151131

[13] Jeong J, Kang HM, Lee EK, Song BM, Kwon YK, Kim HR, et al Highly pathogenic avian influenza virus (H5N8) in domestic poultry and its relationship with migratory birds in South Korea during 2014. Veterinary microbiology. 2014;173(3–4):249–57. doi: 10.1016/j.vetmic.2014.08.002 . 25192767 10.1016/j.vetmic.2014.08.002

[14] Saito T, Tanikawa T, Uchida Y, Takemae N, Kanehira K, Tsunekuni R. Intracontinental and intercontinental dissemination of Asian H5 highly pathogenic avian influenza virus (clade 2.3.4.4) in the winter of 2014–2015. Rev Med Virol. 2015;25(6):388–405. doi: 10.1002/rmv.1857 . 26458727 10.1002/rmv.1857

[15] Schaal T. 2015 USA highly pathogenic avian influenza outbreak review and lessons learned Australian Poultry Science Symposium; Sydney 2016 p. 190–5.

[16] Health OWOfA. OIE Situation Report for Avian Influenza. 2017 10/07/2017. Report No.

[17] Hamilton S. Simulating the transmission and control of highly pathogenic avian influenza in the Australian commercial poultry industries [PhD]: University of Sydney; 2011.

[18] AECL. Australian Egg Corporation Limited Annual Report 2016. 2016.

[19] Grillo VL, Arzey KE, Hansbro PM, Hurt AC, Warner S, Bergfeld J, et al Avian influenza in Australia: a summary of 5 years of wild bird surveillance. Australian veterinary journal. 2015;93(11):387–93. doi: 10.1111/avj.12379 . 26503532 10.1111/avj.12379

[20] Arzey G. The role of wild waterfowl in the epidemiology of AI in Australia. Australian veterinary journal. 2005;83(7):445 . 16035188 10.1111/j.1751-0813.2005.tb13090.x

[21] Swayne DE, Swayne D. Epidemiology of avian influenza in agricultural and other man-made systems. Avian influenza. 2008:59–85.

[22] Selleck PW, Gleeson LJ, Hooper PT, Westbury HA, Hansson E. Identification and characterisation of an H7N3 influenza A virus from an outbreak of virulent avian influenza in Victoria. Australian veterinary journal. 1997;75(4):289–92. . 9140656 10.1111/j.1751-0813.1997.tb10099.x

[23] Selleck PW, Arzey G, Kirkland PD, Reece RL, Gould AR, Daniels PW, et al An outbreak of highly pathogenic avian influenza in Australia in 1997 caused by an H7N4 virus. Avian diseases. 2003;47(3 Suppl):806–11. doi: 10.1637/0005-2086-47.s3.806 . 14575068 10.1637/0005-2086-47.s3.806

[24] Hansbro PM, Warner S, Tracey JP, Arzey KE, Selleck P, O'Riley K, et al Surveillance and analysis of avian influenza viruses, Australia. Emerging infectious diseases. 2010;16(12):1896–904. doi: 10.3201/eid1612.100776 ; PubMed Central PMCID: PMCPMC3294589. 21122219 10.3201/eid1612.100776 PMC3294589

[25] Suarez DL, Swayne D. Influenza A virus. Avian influenza. 2008;1:3–22.

[26] Hamilton S, East I, Toribio JA, Garner M. Are the Australian poultry industries vulnerable to large outbreaks of highly pathogenic avian influenza? Australian veterinary journal. 2009;87(5):165–74. doi: 10.1111/j.1751-0813.2009.00423.x . 19382922 10.1111/j.1751-0813.2009.00423.x

[27] Stebler N, Schuepbach-Regula G, Braam P, Falzon LC. Use of a modified Delphi panel to identify and weight criteria for prioritization of zoonotic diseases in Switzerland. Preventive veterinary medicine. 2015;121(1–2):165–9. doi: 10.1016/j.prevetmed.2015.05.006 . 26036342 10.1016/j.prevetmed.2015.05.006

[28] Kuster K, Cousin ME, Jemmi T, Schupbach-Regula G, Magouras I. Expert Opinion on the Perceived Effectiveness and Importance of On-Farm Biosecurity Measures for Cattle and Swine Farms in Switzerland. PloS one. 2015;10(12):e0144533 doi: 10.1371/journal.pone.0144533 ; PubMed Central PMCID: PMCPMC4686079. 26656893 10.1371/journal.pone.0144533 PMC4686079

[29] Speirs‐Bridge A, Fidler F, McBride M, Flander L, Cumming G, Burgman M. Reducing overconfidence in the interval judgments of experts. Risk Analysis. 2010;30(3):512–23. doi: 10.1111/j.1539-6924.2009.01337.x 20030766 10.1111/j.1539-6924.2009.01337.x

[30] Gigerenzer G. Reckoning with risk: learning to live with uncertainty: Allen Lane The Penguin Press; 2002. 320 p.

[31] Australia B. Importation of Fresh Bananas from the Philippines: Draft IRA Report Department of Agriculture, Fisheries and Forestry, Canberra 2002.

[32] Vose D. Risk analysis: a quantitative guide: John Wiley & Sons; 2008.

[33] Selleck PW, Arzey G, Kirkland PD, Reece RL, Gould AR, Daniels PW, et al An outbreak of highly pathogenic avian influenza in Australia in 1997 caused by an H7N4 virus. Avian diseases. 2003;47:806–11. doi: 10.1637/0005-2086-47.s3.806 PubMed PMID: WOS:000185516000007. 14575068 10.1637/0005-2086-47.s3.806

[34] Berhane Y, Hisanaga T, Kehler H, Neufeld J, Manning L, Argue C, et al Highly pathogenic avian influenza virus A (H7N3) in domestic poultry, Saskatchewan, Canada, 2007. Emerging infectious diseases. 2009;15(9):1492–5. doi: 10.3201/eid1509.080231 ; PubMed Central PMCID: PMCPMC2819867. 19788823 10.3201/eid1509.080231 PMC2819867

[35] Sivanandan V, Halvorson DA, Laudert E, Senne DA, Kumar MC. Isolation of H13N2 influenza A virus from turkeys and surface water. Avian diseases. 1991;35(4):974–7. . 1838479

[36] Marangon S, Capua I. Control of avian influenza in Italy: from stamping out to emergency and prophylactic vaccination. Developments in biologicals. 2006;124:109–15. . 16447501

[37] Nishiguchi A, Yamamoto T, Tsutsui T, Sugizaki T, Mase M, Tsukamoto K, et al Control of an outbreak of highly pathogenic avian influenza, caused by the virus sub-type H5N1, in Japan in 2004. Revue scientifique et technique. 2005;24(3):933–44. . 16642763

[38] Hansbro PM, Warner S, Tracey JP, Arzey KE, Selleck P, O’Riley K, et al Surveillance and analysis of avian influenza viruses, Australia. Emerging infectious diseases. 2010;16(12):1896–904. doi: 10.3201/eid1612.100776 21122219 10.3201/eid1612.100776 PMC3294589

[39] Hamilton SA, East IJ, Toribio JA, Garner MG. Are the Australian poultry industries vulnerable to large outbreaks of highly pathogenic avian influenza? Australian veterinary journal. 2009;87(5):165–74. doi: 10.1111/j.1751-0813.2009.00423.x . 19382922 10.1111/j.1751-0813.2009.00423.x

[40] Markwell DD, Shortridge KF. Possible waterborne transmission and maintenance of influenza viruses in domestic ducks. Applied and environmental microbiology. 1982;43(1):110–5. ; PubMed Central PMCID: PMC241789. 7055370 10.1128/aem.43.1.110-115.1982 PMC241789

[41] Shigematsu S, Dublineau A, Sawoo O, Batejat C, Matsuyama T, Leclercq I, et al Influenza A virus survival in water is influenced by the origin species of the host cell. Influenza and other respiratory viruses. 2014;8(1):123–30. doi: 10.1111/irv.12179 ; PubMed Central PMCID: PMCPMC4177806. 24112132 10.1111/irv.12179 PMC4177806

[42] Zhang H, Li Y, Chen J, Chen Q, Chen Z. Perpetuation of H5N1 and H9N2 avian influenza viruses in natural water bodies. Journal of General Virology. 2014;95(7):1430–5. doi: 10.1099/vir.0.063438–0 24671753 10.1099/vir.0.063438-0

[43] Henaux V, Samuel MD. Avian influenza shedding patterns in waterfowl: implications for surveillance, environmental transmission, and disease spread. Journal of wildlife diseases. 2011;47(3):566–78. doi: 10.7589/0090-3558-47.3.566 . 21719821 10.7589/0090-3558-47.3.566

[44] Zhang H, Xu B, Chen Q, Chen J, Chen Z. Characterization of an H10N8 influenza virus isolated from Dongting lake wetland. Virology journal. 2011;8:42 doi: 10.1186/1743-422X-8-42 ; PubMed Central PMCID: PMCPMC3038951. 21272297 10.1186/1743-422X-8-42 PMC3038951

[45] Shoham D, Jahangir A, Ruenphet S, Takehara K. Persistence of avian influenza viruses in various artificially frozen environmental water types. Influenza Res Treat. 2012;2012:912326 doi: 10.1155/2012/912326 ; PubMed Central PMCID: PMCPMC3471417. 23091712 10.1155/2012/912326 PMC3471417

[46] Guerrini L, Paul MC, Leger L, Andriamanivo HR, Maminiaina OF, Jourdan M, et al Landscape attributes driving avian influenza virus circulation in the Lake Alaotra region of Madagascar. Geospatial health. 2014;8(2):445–53. doi: 10.4081/gh.2014.33 . 24893021 10.4081/gh.2014.33

[47] Zhang H, Li Y, Chen J, Chen Q, Chen Z. Perpetuation of H5N1 and H9N2 avian influenza viruses in natural water bodies. The Journal of general virology. 2014;95(Pt 7):1430–5. doi: 10.1099/vir.0.063438-0 . 24671753 10.1099/vir.0.063438-0

[48] Berhane Y, Ojkic D, Neufeld J, Leith M, Hisanaga T, Kehler H, et al Molecular characterization of pandemic H1N1 influenza viruses isolated from turkeys and pathogenicity of a human pH1N1 isolate in turkeys. Avian diseases. 2010;54(4):1275–85. doi: 10.1637/9422-061410-Reg.1 . 21313850 10.1637/9422-061410-Reg.1

[49] Dunn PA, Wallner-Pendleton EA, Lu H, Shaw DP, Kradel D, Henzler DJ, et al Summary of the 2001–02 Pennsylvania H7N2 low pathogenicity avian influenza outbreak in meat type chickens. Avian diseases. 2003;47(3 Suppl):812–6. doi: 10.1637/0005-2086-47.s3.812 . 14575069 10.1637/0005-2086-47.s3.812

[50] Bowes VA, Ritchie SJ, Byrne S, Sojonky K, Bidulka JJ, Robinson JH. Virus characterization, clinical presentation, and pathology associated with H7N3 avian influenza in British Columbia broiler breeder chickens in 2004. Avian diseases. 2004;48(4):928–34. doi: 10.1637/7218-060304R . 15666877 10.1637/7218-060304R

[51] Spickler AR, Trampel DW, Roth JA. The onset of virus shedding and clinical signs in chickens infected with high-pathogenicity and low-pathogenicity avian influenza viruses. Avian pathology: journal of the WVPA. 2008;37(6):555–77. doi: 10.1080/03079450802499118 . 19023755 10.1080/03079450802499118

[52] Spackman E, Gelb J Jr., Preskenis LA, Ladman BS, Pope CR, Pantin-Jackwood MJ, et al The pathogenesis of low pathogenicity H7 avian influenza viruses in chickens, ducks and turkeys. Virology journal. 2010;7:331 doi: 10.1186/1743-422X-7-331 ; PubMed Central PMCID: PMCPMC3002305. 21092107 10.1186/1743-422X-7-331 PMC3002305

[53] Rebel JM, Peeters B, Fijten H, Post J, Cornelissen J, Vervelde L. Highly pathogenic or low pathogenic avian influenza virus subtype H7N1 infection in chicken lungs: small differences in general acute responses. Veterinary research. 2011;42:10 doi: 10.1186/1297-9716-42-10 ; PubMed Central PMCID: PMCPMC3037890. 21314972 10.1186/1297-9716-42-10 PMC3037890

[54] Comin A, Stegeman A, Marangon S, Klinkenberg D. Evaluating surveillance strategies for the early detection of low pathogenicity avian influenza infections. PloS one. 2012;7(4):e35956 doi: 10.1371/journal.pone.0035956 ; PubMed Central PMCID: PMCPMC3335804. 22545151 10.1371/journal.pone.0035956 PMC3335804

[55] Kapczynski DR, Pantin-Jackwood M, Guzman SG, Ricardez Y, Spackman E, Bertran K, et al Characterization of the 2012 highly pathogenic avian influenza H7N3 virus isolated from poultry in an outbreak in Mexico: pathobiology and vaccine protection. Journal of virology. 2013;87(16):9086–96. doi: 10.1128/JVI.00666-13 ; PubMed Central PMCID: PMCPMC3754080. 23760232 10.1128/JVI.00666-13 PMC3754080

[56] Marche S, Van Borm S, Lambrecht B, Houdart P, van den Berg T. Chasing notifiable avian influenza in domestic poultry: a case report of low-pathogenic avian influenza h5 viruses in two Belgian holdings. Transboundary and emerging diseases. 2014;61(6):526–36. doi: 10.1111/tbed.12056 . 23347839 10.1111/tbed.12056

[57] Bano S, Naeem K, Malik SA. Evaluation of pathogenic potential of avian influenza virus serotype H9N2 in chickens. Avian diseases. 2003;47(3 Suppl):817–22. doi: 10.1637/0005-2086-47.s3.817 . 14575070 10.1637/0005-2086-47.s3.817

[58] Gonzales JL, Boender GJ, Elbers AR, Stegeman JA, de Koeijer AA. Risk based surveillance for early detection of low pathogenic avian influenza outbreaks in layer chickens. Preventive veterinary medicine. 2014;117(1):251–9. doi: 10.1016/j.prevetmed.2014.08.015 . 25217408 10.1016/j.prevetmed.2014.08.015

[59] Kao RR, Haydon DT, Lycett SJ, Murcia PR. Supersize me: how whole-genome sequencing and big data are transforming epidemiology. Trends in microbiology. 2014;22(5):282–91. doi: 10.1016/j.tim.2014.02.011 24661923 10.1016/j.tim.2014.02.011 PMC7125769

[60] Kilpatrick AM, Chmura AA, Gibbons DW, Fleischer RC, Marra PP, Daszak P. Predicting the global spread of H5N1 avian influenza. Proceedings of the National Academy of Sciences of the United States of America. 2006;103(51):19368–73. doi: 10.1073/pnas.0609227103 ; PubMed Central PMCID: PMCPMC1748232. 17158217 10.1073/pnas.0609227103 PMC1748232

[61] Hop GE, Saatkamp HW. A PathWayDiagram for introduction and prevention of Avian Influenza: Application to the Dutch poultry sector. Preventive veterinary medicine. 2010;97(3–4):270–3. doi: 10.1016/j.prevetmed.2010.09.016 . 20950877 10.1016/j.prevetmed.2010.09.016

[62] Yoon H, Park CK, Nam HM, Wee SH. Virus spread pattern within infected chicken farms using regression model: the 2003–2004 HPAI epidemic in the Republic of Korea. Journal of veterinary medicine B, Infectious diseases and veterinary public health. 2005;52(10):428–31. doi: 10.1111/j.1439-0450.2005.00891.x . 16364017 10.1111/j.1439-0450.2005.00891.x

[63] Bos ME, Van Boven M, Nielen M, Bouma A, Elbers AR, Nodelijk G, et al Estimating the day of highly pathogenic avian influenza (H7N7) virus introduction into a poultry flock based on mortality data. Veterinary research. 2007;38(3):493–504. doi: 10.1051/vetres:2007008 . 17425936 10.1051/vetres:2007008

[64] Bataille A, van der Meer F, Stegeman A, Koch G. Evolutionary analysis of inter-farm transmission dynamics in a highly pathogenic avian influenza epidemic. PLoS pathogens. 2011;7(6):e1002094 doi: 10.1371/journal.ppat.1002094 ; PubMed Central PMCID: PMCPMC3121798. 21731491 10.1371/journal.ppat.1002094 PMC3121798

[65] Edmunds KL, Hunter PR, Few R, Bell DJ. Hazard analysis of critical control points assessment as a tool to respond to emerging infectious disease outbreaks. PloS one. 2013;8(8):e72279 doi: 10.1371/journal.pone.0072279 ; PubMed Central PMCID: PMC3743774. 23967294 10.1371/journal.pone.0072279 PMC3743774

[66] Thomas ME, Bouma A, Ekker HM, Fonken AJM, Stegeman JA, Nielen M. Risk factors for the introduction of high pathogenicity Avian Influenza virus into poultry farms during the epidemic in the Netherlands in 2003. Preventive veterinary medicine. 2005;69(1–2):1–11. doi: 10.1016/j.prevetmed.2004.12.001 15899292 10.1016/j.prevetmed.2004.12.001

[67] Nishiguchi A, Kobayashi S, Ouchi Y, Yamamoto T, Hayama Y, Tsutsui T. Spatial Analysis of Low Pathogenic H5N2 Avian Influenza Outbreaks in Japan in 2005. Journal of Veterinary Medical Science. 2009;71(7):979–82. doi: 10.1292/jvms.71.979 19652489 10.1292/jvms.71.979

[68] Wakawa AM, Abdu P, Oladele S, Sa’idu L, Mohammed S. Risk factors for the occurrence and spread of Highly Pathogenic Avian Influenza H5N1 in commercial poultry farms in Kano, Nigeria. Sokoto Journal of Veterinary Sciences. 2012;10(2):40–51.

