# Correction: Assessing the probability of introduction and spread of avian influenza (AI) virus in commercial Australian poultry operations using an expert opinion elicitation

**DOI:** 10.1371/journal.pone.0212895

**Published:** 2019-02-21

**Authors:** Mini Singh, Jenny-Ann Toribio, Angela Bullanday Scott, Peter Groves, Belinda Barnes, Kathryn Glass, Barbara Moloney, Amanda Black, Marta Hernandez-Jover

The first sentence of the second paragraph of the Introduction should read: Australia has had seven highly pathogenic avian influenza (HPAI) outbreaks on chicken farms during the last 42 years (1976–2018), two of which have occurred in the last 10 years.

Reference 20 should read: Office of Chief Veterinary Officer (AU). National Avian Influenza Surveillance Dossier. Canberra: Australian Government Department of Agriculture, Fisheries and Forestry; 2010. 165 p. [2]

The last sentence of the fifth paragraph of the Introduction should read: Six of the seven outbreaks occurred in poultry low density regions (as outlined in farm density maps [26]) and the potential for spread of HPAI between farms in higher density regions is also not known [26].

A reference is omitted from the last sentence of the second paragraph of the Discussion section, included to account for the 2012 and 2013 outbreaks.

The sentence should read: In the seven HPAI out-breaks reported in poultry in Australia, introduction of LPAI viruses from wild birds and subsequent mutation has been hypothesized as the most likely origin of these outbreaks [19, 38, 39 (Brown et al. 2016)].

The reference is: Brown I, Abolnik C, Garcia-Garcia J, McCullough S, Swayne DE, Cattoli G. High‐pathogenicity avian influenza outbreaks since 2008, excluding multi‐continental panzootic of H5 Goose/Guangdong‐lineage viruses. In: Swayne DE, editor. Animal Influenza 2nd ed. New York City: USA: Wiley; 2016. pp. 248–270. [3]
