# Supplementary material for: Assessing the probability of introduction and spread of avian influenza (AI) virus in commercial Australian poultry operations using an expert opinion elicitation
Source: PLoS One. 2018 Mar 1;13(3):e0193730. doi: 10.1371/journal.pone.0193730 (PMC5832321; doi:10.1371/journal.pone.0193730)
Supplement: S1 Appendix — (DOCX) [file pone.0193730.s001.docx]

## EXPERT CONSULTATION ON PATHWAYS OF INTRODUCTION AND SPREAD OF AVIAN INFLUENZA INTO COMMERCIAL POULTRY OPERATIONS IN NSW

**Name:**

**Organization:**

**Years of experience in the poultry industry:**

This questionnaire and workshop are intended to gain information from your unique knowledge base. We are using the methodology for acquiring estimates and we will use the group averages for our subsequent assessments.

For this reason, it is very important that you answer on the basis of your best knowledge. There is absolutely no need for you to look for the answers online or in books. We just want the information based on your experience and thus informed opinions that you have acquired uniquely over so many years.

For this methodology **it is crucial that we receive your answers to this questionnaire before Thursday, October 15**, as the workshop discussions will be based on these answers. If you have any questions or concerns, please contact me.

Mini Singh

Ph: 02 9351 1639 / 0451 669 479

Email: mini.singh@sydney.edu.au

## Background information

For this assessment, questions regarding pathways and probabilities of introduction and spread of avian influenza virus into a commercial chicken layer or broiler operation should be answered ***considering that Low Pathogenic Avian Influenza (LPAI) is already prevalent in some wild birds in Australia,*** including H5 and H7.

**Highly Pathogenic Avian Influenza outbreaks in Australia**:

*There have been seven HPAI outbreaks reported in Australia since 1976, all involving non-exotic H7 viruses and causing clinical disease on the affected commercial chicken farms. For the five outbreaks that occurred between 1976 and 1997, there was evidence of the chickens having direct contact with wild waterfowl or indirect contact via contaminated surface water, or contact with free-range farmed ducks (*[*Hamilton et al., 2009*](#_ENREF_20)*,* [*DAFF-Queensland, 2013*](#_ENREF_9)*,* [*Selleck et al., 2003*](#_ENREF_36)*,* [*Hansbro et al., 2010*](#_ENREF_21)*,* [*Tracey et al., 2004*](#_ENREF_38)*,* [*Forsyth et al., 1993*](#_ENREF_15)*,* [*Forman et al., 1986*](#_ENREF_14)*,* [*Morgan and Kelly, 1990*](#_ENREF_29)*,* [*Arzey, 2005*](#_ENREF_4)*,* [*Turner, 2004*](#_ENREF_40)*). For the two recent outbreaks in New South Wales, all or some of the layer flock on each farm were under free-range management. Secondary spread of the infection from the index property occurred in three of these outbreaks through fomites, although this spread was limited. Pathways for secondary spread implicated were an employee who owned a duck farm for the 1992 outbreak in Bendigo, a dead bird collector contractor for the 1997 outbreak in Tamworth and re-used cardboard egg cartons for the 2013 outbreak in Young (Selleck, 2003, DAFF Queensland, 2013)*

For the purpose of this assessment **commercial broiler operations are operations with > 50,000 broilers.**

For the purpose of this assessment **commercial layer operations are operations with > 1000 laying hens.**

**Questionnaire:**

- This questionnaire is organized in four different sections with questions referring to:
  - ***LPAI introduction into a shed***
  - ***LPAI established in a shed***
  - ***Spread shed-to-shed on a farm***
  - ***Spread farm-to-farm***
- Most questions refer to a set of 100 typical operations and your answers should provide a number from 0 to 100.

- It may help you, when answering the questions, to use the table below as a rough guide (equivalent to Biosecurity Australia terminology):

| **Qualitative Probability** | **Natural frequencies**  ***The number of cases / events / operations out of 100*** |
| --- | --- |
| High  Moderate  Low  Very low | 70 - 100  30 – 70  5 - 30  0.1 - 5 |

## Methodology

##

For questions about the frequency of events, we will use what is called a 4-step format. The diagram below gives the general form of the questions, showing how they relate to a ‘confidence interval’, which includes your best guess and a region around it, that reflects how certain you are about your answer (see ACERA Project 0611).

4. How confident are you that the interval you created, from lowest to highest, will capture the true value? Please enter a number between 50 and 100%

1. Realistically, what do you think the lowest number could be?

2. Realistically, what do you think the highest number could be?

3. Realistically, what is the most likely number?

- ***Step 1 and 2:*** *Lowest and highest number operations:*

These questions ask you to estimate the lowest and the highest plausible number of operations, setting the limits for the interval that you believe encloses the truth.

- ***Step 3:*** *Most likely number of operations:*

These questions ask you to provide your best guess for the true number of operations.

- ***Step 4:*** *Confidence:*

The last column of the tables asks you to provide a percentage that represents how confident (how certain) you are that the true value falls within the interval you created from 50 to 100% (If your confidence is less than 50% means that you believe that your best guess will fall outside your estimate interval in most of the cases).

- The word ‘***Realistically***’ is used for you to think about **plausible numbers** for your estimations (instead of possible extreme numbers).

Your participation on this exercise is very much appreciated by all the members of the research team.

Thank you very much!

1. How often do you deal with **commercial poultry operations**?

|  |
| --- |

1. Which **commercial poultry operations** are you involved with?

|  |
| --- |

1. What is your involvement with these **commercial poultry operations**?

|  |
| --- |

1. To the best of your knowledge, how many **commercial* free range layer** farms are operational in NSW? (* **commercial** layer operations > 1000 laying hens)

|  |
| --- |

1. To the best of your knowledge, how many **commercial* free range broiler** farms are operational in NSW? (***commercial** broiler operations > 50,000 broilers)

|  |
| --- |

**LPAI INTRODUCTION INTO A SHED**

1. What is the prevalence of **LPAI** in wild bird populations in Australia?

|  |
| --- |

WILD BIRD PATHWAYS

Imagine 100 **broiler barn sheds** where LPAI has recently been introduced. Realistically how many of these would have experienced an **LPAI** introduction to the shed through each of the following pathways?

|  | Realistically, what do you think the lowest number of sheds would be? | Realistically, what do you think the highest number of sheds would be? | Realistically, what do you think the most likely number of sheds would be? | How confident (50 – 100%) are you that this interval will include the true value? |
| --- | --- | --- | --- | --- |
| Direct contact with wild bird |  |  |  |  |
| Indirect contact with wild bird through a contaminated water source |  |  |  |  |
| Indirect contact with wild bird through faeces in the shed |  |  |  |  |
| Indirect contact with wild bird through aerial dispersion of faeces in dust (wind, fans, endotoxins, etc…) |  |  |  |  |

Imagine 100 **free range broiler sheds** where LPAI has recently been introduced. Realistically how many of these would have experienced an **LPAI** introduction to the shed through each of the following pathways?

|  | Realistically, what do you think the lowest number of sheds would be? | Realistically, what do you think the highest number of sheds would be? | Realistically, what do you think the most likely number of sheds would be? | How confident (50 – 100%) are you that this interval will include the true value? |
| --- | --- | --- | --- | --- |
| Direct contact with wild bird |  |  |  |  |
| Indirect contact with wild bird through a contaminated water source |  |  |  |  |
| Indirect contact with wild bird through faeces in the shed/range area |  |  |  |  |
| Indirect contact with wild bird through aerial dispersion of faeces in dust (wind, fans, endotoxins, etc…) |  |  |  |  |

Imagine 100 **cage layer sheds** where LPAI has recently been introduced. Realistically how many of these would have experienced an **LPAI** introduction to the shed through each of the following pathways?

|  | Realistically, what do you think the lowest number of sheds would be? | Realistically, what do you think the highest number of sheds would be? | Realistically, what do you think the most likely number of sheds would be? | How confident (50 – 100%) are you that this interval will include the true value? |
| --- | --- | --- | --- | --- |
| Direct contact with wild bird |  |  |  |  |
| Indirect contact with wild bird through a contaminated water source |  |  |  |  |
| Indirect contact with wild bird through faeces in the shed |  |  |  |  |
| Indirect contact with wild bird through aerial dispersion of faeces in dust (wind, fans, endotoxins, etc…) |  |  |  |  |

Imagine 100 **barn layer sheds** where LPAI has recently been introduced. Realistically how many of these would have experienced an **LPAI i**ntroduction to the shed through each of the following pathways?

|  | Realistically, what do you think the lowest number of sheds would be? | Realistically, what do you think the highest number of sheds would be? | Realistically, what do you think the most likely number of sheds would be? | How confident (50 – 100%) are you that this interval will include the true value? |
| --- | --- | --- | --- | --- |
| Direct contact with wild bird |  |  |  |  |
| Indirect contact with wild bird through a contaminated water source |  |  |  |  |
| Indirect contact with wild bird through faeces in the shed |  |  |  |  |
| Indirect contact with wild bird through aerial dispersion of faeces in dust (wind, fans, endotoxins, etc…) |  |  |  |  |

Imagine 100 **free range layer sheds** where LPAI has recently been introduced. Realistically how many of these would have experienced an **LPAI** introduction to the shed through each of the following pathways?

|  | Realistically, what do you think the lowest number of sheds would be? | Realistically, what do you think the highest number of sheds would be? | Realistically, what do you think the most likely number of sheds would be? | How confident (50 – 100%) are you that this interval will include the true value? |
| --- | --- | --- | --- | --- |
| Direct contact with wild bird |  |  |  |  |
| Indirect contact with wild bird through a contaminated water source |  |  |  |  |
| Indirect contact with wild bird through faeces in the shed/range area |  |  |  |  |
| Indirect contact with wild bird through aerial dispersion of faeces in dust (wind, fans, endotoxins, etc…) |  |  |  |  |

DISTANCE FROM UNPROTECTED WATER BODY

Imagine an unprotected water body source that results in the congregation of wild birds, say 50% of which are infected with LPAI. If there were 100 **broiler barn sheds** located at each of the specified distances from the water body listed below, realistically how many of them will experience an **LPAI** introduction?

|  | Realistically, what do you think the lowest number of sheds would be? | Realistically, what do you think the highest number of sheds would be? | Realistically, what do you think the most likely number of sheds would be? | How confident (50 – 100%) are you that this interval will include the true value? |
| --- | --- | --- | --- | --- |
| Water body >1km from the shed |  |  |  |  |
| Water body between 500m - 1km from the shed |  |  |  |  |
| Water body between 100m - 500m from the shed |  |  |  |  |
| Water body <100m from the shed |  |  |  |  |

Imagine an unprotected water body source that results in the congregation of wild birds, say 50% of which are infected with LPAI. If there were 100 **free range** **broiler sheds** with their perimeter fence of range located at each of the specified distances from the water body listed below, realistically how many of them will experience an **LPAI** introduction?

|  | Realistically, what do you think the lowest number of sheds would be? | Realistically, what do you think the highest number of sheds would be? | Realistically, what do you think the most likely number of sheds would be? | How confident (50 – 100%) are you that this interval will include the true value? |
| --- | --- | --- | --- | --- |
| Water body >1km from the perimeter fence of range |  |  |  |  |
| Water body between 500m - 1km from the perimeter fence of range |  |  |  |  |
| Water body between 100m - 500m from the perimeter fence of range |  |  |  |  |
| Water body <100m from the perimeter fence of range |  |  |  |  |

Imagine an unprotected water body source that results in the congregation of wild birds, say 50% of which are infected with LPAI. If there were 100 **cage layer sheds** located at each of the specified distances from the water body listed below, realistically how many of them will experience an **LPAI** introduction?

|  | Realistically, what do you think the lowest number of sheds would be? | Realistically, what do you think the highest number of sheds would be? | Realistically, what do you think the most likely number of sheds would be? | How confident (50 – 100%) are you that this interval will include the true value? |
| --- | --- | --- | --- | --- |
| Water body >1km from the shed |  |  |  |  |
| Water body between 500m - 1km from the shed |  |  |  |  |
| Water body between 100m - 500m from the shed |  |  |  |  |
| Water body <100m from the shed |  |  |  |  |

Imagine an unprotected water body source that results in the congregation of wild birds, say 50% of which are infected with LPAI. If there were 100 **barn layer sheds** located at each of the specified distances from the water body listed below, realistically how many of them will experience an **LPAI** introduction?

|  | Realistically, what do you think the lowest number of sheds would be? | Realistically, what do you think the highest number of sheds would be? | Realistically, what do you think the most likely number of sheds would be? | How confident (50 – 100%) are you that this interval will include the true value? |
| --- | --- | --- | --- | --- |
| Water body >1km from the shed |  |  |  |  |
| Water body between 500m - 1km from the shed |  |  |  |  |
| Water body between 100m - 500m from the shed |  |  |  |  |
| Water body <100m from the shed |  |  |  |  |

Imagine an unprotected water body source that results in the congregation of wild birds, say 50% of which are infected with LPAI. If there were 100 **free range** **layer sheds** with their perimeter fence of range located at each of the specified distances from the water body listed below, realistically how many of them will experience an **LPAI** introduction?

|  | Realistically, what do you think the lowest number of sheds would be? | Realistically, what do you think the highest number of sheds would be? | Realistically, what do you think the most likely number of sheds would be? | How confident (50 – 100%) are you that this interval will include the true value? |
| --- | --- | --- | --- | --- |
| Water body >1km from the perimeter fence of range |  |  |  |  |
| Water body between 500m - 1km from the perimeter fence of range |  |  |  |  |
| Water body between 100m - 500m from the perimeter fence of range |  |  |  |  |
| Water body <100m from the perimeter fence of range |  |  |  |  |

1. List potential pathways, other than mentioned above, of introduction of notifiable low pathogenic avian influenza (**H7 & H5 LPAI**) into a commercial chicken flock for the following operations (please add more lines if required)

Broiler Barn

1.___________________________

2.___________________________

3.___________________________

4.___________________________

Broiler Free Range

1.___________________________

2.___________________________

3.___________________________

4.___________________________

Layer Cage

1.___________________________

2.___________________________

3.___________________________

4.___________________________

Layer Barn

1.___________________________

2.___________________________

3.___________________________

4.___________________________

Layer Free Range

1.___________________________

2.___________________________

3.___________________________

4.___________________________

**LPAI ESTABLISHED IN A SHED**

Imagine 100 **sheds each of the following operation types** where LPAI has recently been established**. Realistically in how many of these sheds would a change in mortality / morbidity/ production attributable to **LPAI** be detected on-farm within a week?

(** Established in this section means LPAI virus is circulating among birds in the shed)

|  | Realistically, what do you think the lowest number of sheds would be? | Realistically, what do you think the highest number of sheds would be? | Realistically, what do you think the most likely number of sheds would be? | How confident (50 – 100%) are you that this interval will include the true value? |
| --- | --- | --- | --- | --- |
| Broiler barn |  |  |  |  |
| Broiler free range |  |  |  |  |
| Layer cage |  |  |  |  |
| Layer barn |  |  |  |  |
| Layer free range |  |  |  |  |

- What factors would influence the detection of **LPAI** in each of these operation types?

|  |
| --- |

1. Imagine 100 **sheds each of the following operation types** where LPAI has recently been established. In how many of these sheds would **LPAI mutate to HPAI**?

|  | Realistically, what do you think the lowest number of sheds would be? | Realistically, what do you think the highest number of sheds would be? | Realistically, what do you think the most likely number of sheds would be? | How confident (50 – 100%) are you that this interval will include the true value? |
| --- | --- | --- | --- | --- |
| Broiler barn |  |  |  |  |
| Broiler free range |  |  |  |  |
| Layer cage |  |  |  |  |
| Layer barn |  |  |  |  |
| Layer free range |  |  |  |  |

- What factors have you considered in providing the estimates?

|  |
| --- |

**SPREAD SHED-TO-SHED ON A FARM**

1. Imagine 100 farms (with multiple sheds) of the following operation types. If there is one **LPAI** established** shed (that has not been detected) on the property, realistically how many of these farms will experience spread of **LPAI** infection to at least one other shed on the property?

(** Established in this section means AI virus is circulating among birds in the shed)

|  | Realistically, what do you think the lowest number of farms would be? | Realistically, what do you think the highest number of farms would be? | Realistically, what do you think the most likely number of farms would be? | How confident (50 – 100%) are you that this interval will include the true value? |
| --- | --- | --- | --- | --- |
| Broiler barn |  |  |  |  |
| Broiler free range |  |  |  |  |
| Layer cage |  |  |  |  |
| Layer barn |  |  |  |  |
| Layer free range |  |  |  |  |

Imagine 100 **broiler barn** farms (with multiple sheds) with one shed where LPAI is established. Realistically how many of these will experience **LPAI** spread to at least one other shed on the property through each of the following pathways?

|  | Realistically, what do you think the lowest number of farms would be? | Realistically, what do you think the highest number of farms would be? | Realistically, what do you think the most likely number of farms would be? | How confident (50 – 100%) are you that this interval will include the true value? |
| --- | --- | --- | --- | --- |
| Direct contact with wild bird infected via the LPAI established shed |  |  |  |  |
| Other animals/insects  (eg rats, snakes, beetles) |  |  |  |  |
| Shared equipment  (eg buckets, shovels) |  |  |  |  |
| Shared personnel |  |  |  |  |
| Aerial dispersion (dust, feathers, endotoxins, fans) |  |  |  |  |

Imagine 100 **free range broiler** farms (with multiple sheds) with one shed where LPAI is established. Realistically how many of these will experience **LPAI** spread to at least one other shed on the property through each of the following pathways?

|  | Realistically, what do you think the lowest number of farms would be? | Realistically, what do you think the highest number of farms would be? | Realistically, what do you think the most likely number of farms would be? | How confident (50 – 100%) are you that this interval will include the true value? |
| --- | --- | --- | --- | --- |
| Direct contact with wild bird infected via the LPAI established shed |  |  |  |  |
| Other animals/insects  (eg rats, snakes, beetles) |  |  |  |  |
| Shared equipment  (eg buckets, shovels) |  |  |  |  |
| Shared personnel |  |  |  |  |
| Aerial dispersion (dust, feathers, endotoxins, fans) |  |  |  |  |

Imagine 100 **cage layer** farms (with multiple sheds) with one shed where LPAI is established. Realistically how many of these will experience **LPAI** spread to at least one other shed on the property through each of the following pathways?

|  | Realistically, what do you think the lowest number of farms would be? | Realistically, what do you think the highest number of farms would be? | Realistically, what do you think the most likely number of farms would be? | How confident (50 – 100%) are you that this interval will include the true value? |
| --- | --- | --- | --- | --- |
| Direct contact with wild bird infected via the LPAI established shed |  |  |  |  |
| Other animals/insects  (eg rats, snakes, beetles) |  |  |  |  |
| Shared equipment  (eg buckets, shovels) |  |  |  |  |
| Shared personnel |  |  |  |  |
| Aerial dispersion (dust, feathers, endotoxins, fans) |  |  |  |  |

Imagine 100 **barn layer** farms (with multiple sheds) with one shed where LPAI is established. Realistically how many of these will experience **LPAI** spread to at least one other shed on the property through each of the following pathways?

|  | Realistically, what do you think the lowest number of farms would be? | Realistically, what do you think the highest number of farms would be? | Realistically, what do you think the most likely number of farms would be? | How confident (50 – 100%) are you that this interval will include the true value? |
| --- | --- | --- | --- | --- |
| Direct contact with wild bird infected via the LPAI established shed |  |  |  |  |
| Other animals/insects  (eg rats, snakes, beetles) |  |  |  |  |
| Shared equipment  (eg buckets, shovels) |  |  |  |  |
| Shared personnel |  |  |  |  |
| Aerial dispersion (dust, feathers, endotoxins, fans) |  |  |  |  |

Imagine 100 **free range layer** farms (with multiple sheds) with one shed where LPAI is established. Realistically how many of these will experience **LPAI** spread to at least one other shed on the property through each of the following pathways?

|  | Realistically, what do you think the lowest number of farms would be? | Realistically, what do you think the highest number of farms would be? | Realistically, what do you think the most likely number of farms would be? | How confident (50 – 100%) are you that this interval will include the true value? |
| --- | --- | --- | --- | --- |
| Direct contact with wild bird infected via the LPAI established shed |  |  |  |  |
| Other animals/insects  (eg rats, snakes, beetles) |  |  |  |  |
| Shared equipment  (eg buckets, shovels) |  |  |  |  |
| Shared personnel |  |  |  |  |
| Aerial dispersion (dust, feathers, endotoxins, fans) |  |  |  |  |

1. Imagine 100 farms (with multiple sheds) of the following operation types. If there is one **HPAI** established shed (that has not been detected) on the property, realistically how many of these farms will experience spread of **HPAI** infection to at least one other shed on the property?

|  | Realistically, what do you think the lowest number of farms would be? | Realistically, what do you think the highest number of farms would be? | Realistically, what do you think the most likely number of farms would be? | How confident (50 – 100%) are you that this interval will include the true value? |
| --- | --- | --- | --- | --- |
| Broiler barn |  |  |  |  |
| Broiler free range |  |  |  |  |
| Layer cage |  |  |  |  |
| Layer barn |  |  |  |  |
| Layer free range |  |  |  |  |

**SPREAD FARM-TO-FARM**

1. Imagine 100 **LPAI** established** farms (where LPAI has not been detected) of the following operation types. Realistically how many of these will experience spread of **LPAI** infection to at least one other chicken farm of any operation type?

(** Established in this section means AI virus is circulating among birds in ≥1 sheds on the farm)

|  | Realistically, what do you think the lowest number of farms would be? | Realistically, what do you think the highest number of farms would be? | Realistically, what do you think the most likely number of farms would be? | How confident (50 – 100%) are you that this interval will include the true value? |
| --- | --- | --- | --- | --- |
| Broiler barn |  |  |  |  |
| Broiler free range |  |  |  |  |
| Layer cage |  |  |  |  |
| Layer barn |  |  |  |  |
| Layer free range |  |  |  |  |

Imagine 100 LPAI established **broiler barn** farms. Realistically how many of these will experience **LPAI** spread to at least one other chicken farm through each of the following pathways.

|  | Realistically, what do you think the lowest number of farms would be? | Realistically, what do you think the highest number of farms would be? | Realistically, what do you think the most likely number of farms would be? | How confident (50 – 100%) are you that this interval will include the true value? |
| --- | --- | --- | --- | --- |
| Wind borne mechanisms |  |  |  |  |
| Direct contact with wild bird that got infected on the farm |  |  |  |  |
| Other animals/insects (eg rats/snakes/beetles) |  |  |  |  |
| Shared bird delivery transport |  |  |  |  |
| Shared bird pick up transport |  |  |  |  |
| Shared feed delivery transport |  |  |  |  |
| Shared manure collection transport |  |  |  |  |
| Shared farm workers |  |  |  |  |
| Shared trades persons (eg electrician/plumber) |  |  |  |  |
| Shared equipment  (eg bobcat/litter mover) |  |  |  |  |

Imagine 100 LPAI established **free range** **broiler** farms. Realistically how many of these will experience **LPAI** spread to at least one other chicken farm through each of the following pathways.

|  | Realistically, what do you think the lowest number of farms would be? | Realistically, what do you think the highest number of farms would be? | Realistically, what do you think the most likely number of farms would be? | How confident (50 – 100%) are you that this interval will include the true value? |
| --- | --- | --- | --- | --- |
| Wind borne mechanisms |  |  |  |  |
| Direct contact with wild bird that got infected on the farm |  |  |  |  |
| Other animals/insects (eg rats/snakes/beetles) |  |  |  |  |
| Shared bird delivery transport |  |  |  |  |
| Shared bird pick up transport |  |  |  |  |
| Shared feed delivery transport |  |  |  |  |
| Shared manure collection transport |  |  |  |  |
| Shared farm workers |  |  |  |  |
| Shared trades persons (eg electrician/plumber) |  |  |  |  |
| Shared equipment  (eg bobcat/litter mover) |  |  |  |  |

Imagine 100 LPAI established **cage layer** farms. Realistically how many of these will experience **LPAI** spread to at least one other chicken farm through each of the following pathways.

|  | Realistically, what do you think the lowest number of farms would be? | Realistically, what do you think the highest number of farms would be? | Realistically, what do you think the most likely number of farms would be? | How confident (50 – 100%) are you that this interval will include the true value? |
| --- | --- | --- | --- | --- |
| Wind borne mechanisms |  |  |  |  |
| Direct contact with wild bird that got infected on the farm |  |  |  |  |
| Other animals/insects eg (rats/snakes/beetles) |  |  |  |  |
| Shared bird delivery transport |  |  |  |  |
| Shared bird pick up transport |  |  |  |  |
| Shared feed delivery transport |  |  |  |  |
| Shared manure collection transport |  |  |  |  |
| Shared farm workers |  |  |  |  |
| Shared trades persons (eg electrician/plumber) |  |  |  |  |
| Shared equipment  (eg bobcat/litter mover) |  |  |  |  |
| Shared egg trays |  |  |  |  |
| Shared egg pallets*** |  |  |  |  |

*** Pallets of packed egg trays can be transported from an infected farm to grading facilities on other farms

Imagine 100 LPAI established **barn layer** farms. Realistically how many of these will experience **LPAI** spread to at least one other chicken farm through each of the following pathways.

|  | Realistically, what do you think the lowest number of farms would be? | Realistically, what do you think the highest number of farms would be? | Realistically, what do you think the most likely number of farms would be? | How confident (50 – 100%) are you that this interval will include the true value? |
| --- | --- | --- | --- | --- |
| Wind borne mechanisms |  |  |  |  |
| Direct contact with wild bird that got infected on the farm |  |  |  |  |
| Other animals/insects (eg rats/snakes/beetles) |  |  |  |  |
| Shared bird delivery transport |  |  |  |  |
| Shared bird pick up transport |  |  |  |  |
| Shared feed delivery transport |  |  |  |  |
| Shared manure collection transport |  |  |  |  |
| Shared farm workers |  |  |  |  |
| Shared trades persons (eg electrician/plumber) |  |  |  |  |
| Shared equipment  (eg bobcat/litter mover) |  |  |  |  |
| Shared egg trays |  |  |  |  |
| Shared egg pallets*** |  |  |  |  |

*** Pallets of packed egg trays can be transported from an infected farm to grading facilities on other farms.

Imagine 100 LPAI established **free range layer** farms. Realistically how many of these will experience **LPAI** spread to at least one other chicken farm through each of the following pathways?

|  | Realistically, what do you think the lowest number of farms would be? | Realistically, what do you think the highest number of farms would be? | Realistically, what do you think the most likely number of farms would be? | How confident (50 – 100%) are you that this interval will include the true value? |
| --- | --- | --- | --- | --- |
| Wind borne mechanisms |  |  |  |  |
| Direct contact with wild bird that got infected on the farm |  |  |  |  |
| Other animals/insects (eg rats/snakes/beetles) |  |  |  |  |
| Shared bird delivery transport |  |  |  |  |
| Shared bird pick up transport |  |  |  |  |
| Shared feed delivery transport |  |  |  |  |
| Shared manure collection transport |  |  |  |  |
| Shared farm workers |  |  |  |  |
| Shared trades persons (eg electrician/plumber) |  |  |  |  |
| Shared equipment  (eg bobcat/litter mover) |  |  |  |  |
| Shared egg trays |  |  |  |  |
| Shared egg pallets*** |  |  |  |  |

*** Pallets of packed egg trays can be transported from an infected farm to grading facilities on other farms.

1. Imagine 100 **HPAI** established farms of the following operation types. Realistically how many of these will experience spread of **HPAI** infection to at least one other chicken farm of any operation type?

|  | Realistically, what do you think the lowest number of farms would be? | Realistically, what do you think the highest number of farms would be? | Realistically, what do you think the most likely number of farms would be? | How confident (50 – 100%) are you that this interval will include the true value? |
| --- | --- | --- | --- | --- |
| Broiler barn |  |  |  |  |
| Broiler free range |  |  |  |  |
| Layer cage |  |  |  |  |
| Layer barn |  |  |  |  |
| Layer free range |  |  |  |  |

Imagine 100 **HPAI** established **broiler barn** farms. Realistically how many of these will experience **HPAI** spread to at least one other chicken farm through each of the following pathways.

|  | Realistically, what do you think the lowest number of farms would be? | Realistically, what do you think the highest number of farms would be? | Realistically, what do you think the most likely number of farms would be? | How confident (50 – 100%) are you that this interval will include the true value? |
| --- | --- | --- | --- | --- |
| Wind borne mechanisms |  |  |  |  |
| Direct contact with wild bird that got infected on the farm |  |  |  |  |
| Other animals/insects (eg rats/snakes/beetles) |  |  |  |  |
| Shared bird delivery transport |  |  |  |  |
| Shared bird pick up transport |  |  |  |  |
| Shared feed delivery transport |  |  |  |  |
| Shared manure collection transport |  |  |  |  |
| Shared farm workers |  |  |  |  |
| Shared trades persons (eg electrician/plumber) |  |  |  |  |
| Shared equipment  (eg bobcat/litter mover) |  |  |  |  |

1. Imagine 100 **HPAI** established **free range** **broiler** farms. Realistically how many of these will experience **HPAI** spread to at least one other chicken farm through each of the following pathways.

|  | Realistically, what do you think the lowest number of farms would be? | Realistically, what do you think the highest number of farms would be? | Realistically, what do you think the most likely number of farms would be? | How confident (50 – 100%) are you that this interval will include the true value? |
| --- | --- | --- | --- | --- |
| Wind borne mechanisms |  |  |  |  |
| Direct contact with wild bird that got infected on the farm |  |  |  |  |
| Other animals/insects (eg rats/snakes/beetles) |  |  |  |  |
| Shared bird delivery transport |  |  |  |  |
| Shared bird pick up transport |  |  |  |  |
| Shared feed delivery transport |  |  |  |  |
| Shared manure collection transport |  |  |  |  |
| Shared farm workers |  |  |  |  |
| Shared trades persons (eg electrician/plumber) |  |  |  |  |
| Shared equipment  (eg bobcat/litter mover) |  |  |  |  |

Imagine 100 **HPAI** established **cage layer** farms. Realistically how many of these will experience **HPAI** spread to at least one other chicken farm through each of the following pathways.

|  | Realistically, what do you think the lowest number of farms would be? | Realistically, what do you think the highest number of farms would be? | Realistically, what do you think the most likely number of farms would be? | How confident (50 – 100%) are you that this interval will include the true value? |
| --- | --- | --- | --- | --- |
| Wind borne mechanisms |  |  |  |  |
| Direct contact with wild bird that got infected on the farm |  |  |  |  |
| Other animals/insects (eg rats/snakes/beetles) |  |  |  |  |
| Shared bird delivery transport |  |  |  |  |
| Shared bird pick up transport |  |  |  |  |
| Shared feed delivery transport |  |  |  |  |
| Shared manure collection transport |  |  |  |  |
| Shared farm workers |  |  |  |  |
| Shared trades persons (eg electrician/plumber) |  |  |  |  |
| Shared equipment  (eg bobcat/litter mover) |  |  |  |  |
| Shared egg trays |  |  |  |  |
| Shared egg pallets*** |  |  |  |  |

*** Pallets of packed egg trays can be transported from an infected farm to grading facilities on other farm

Imagine 100 **HPAI** established **barn layer** farms. Realistically how many of these will experience **HPAI** spread to at least one other chicken farm through each of the following pathways.

|  | Realistically, what do you think the lowest number of farms would be? | Realistically, what do you think the highest number of farms would be? | Realistically, what do you think the most likely number of farms would be? | How confident (50 – 100%) are you that this interval will include the true value? |
| --- | --- | --- | --- | --- |
| Wind borne mechanisms |  |  |  |  |
| Direct contact with wild bird that got infected on the farm |  |  |  |  |
| Other animals/insects (eg rats/snakes/beetles) |  |  |  |  |
| Shared bird delivery transport |  |  |  |  |
| Shared bird pick up transport |  |  |  |  |
| Shared feed delivery transport |  |  |  |  |
| Shared manure collection transport |  |  |  |  |
| Shared farm workers |  |  |  |  |
| Shared trades persons (eg electrician/plumber) |  |  |  |  |
| Shared equipment  (eg bobcat/litter mover) |  |  |  |  |
| Shared egg trays |  |  |  |  |
| Shared egg pallets*** |  |  |  |  |

*** Pallets of packed egg trays can be transported from an infected farm to grading facilities on other farms.

Imagine 100 **HPAI** established **free range layer** farms. Realistically how many of these will experience **HPAI** spread to at least one other chicken farm through each of the following pathways?

|  | Realistically, what do you think the lowest number of farms would be? | Realistically, what do you think the highest number of farms would be? | Realistically, what do you think the most likely number of farms would be? | How confident (50 – 100%) are you that this interval will include the true value? |
| --- | --- | --- | --- | --- |
| Wind borne mechanisms |  |  |  |  |
| Direct contact with wild bird that got infected on the farm |  |  |  |  |
| Other animals/insects (eg rats/snakes/beetles) |  |  |  |  |
| Shared bird delivery transport |  |  |  |  |
| Shared bird pick up transport |  |  |  |  |
| Shared feed delivery transport |  |  |  |  |
| Shared manure collection transport |  |  |  |  |
| Shared farm workers |  |  |  |  |
| Shared trades persons (eg electrician/plumber) |  |  |  |  |
| Shared equipment  (eg bobcat/litter mover) |  |  |  |  |
| Shared egg trays |  |  |  |  |
| Shared egg pallets*** |  |  |  |  |

*** Pallets of packed egg trays can be transported from an infected farm to grading facilities on other farms.

1. If there is **one farm** of the following operation types **infected** with notifiable **HPAI**, what pathways other than the ones listed above could spread infection from this farm to other chicken farms? (Please add more lines if required).

Broiler Barn

1.___________________________

2.___________________________

3.___________________________

4.___________________________

Broiler Free Range

1.___________________________

2.___________________________

3.___________________________

4.___________________________

Layer Cage

1.___________________________

2.___________________________

3.___________________________

4.___________________________

Layer Barn

1.___________________________

2.___________________________

3.___________________________

4.___________________________

Layer Free Range

1.___________________________

2.___________________________

3.___________________________

4.___________________________

1. To the best of your knowledge, how many broiler and layer farms in NSW fall under the migratory flyover pathway of wild birds?

|  |
| --- |

1. As a result of the Avian Influenza outbreak and resulting depopulation efforts in the US in 2015, how many poultry have died or been culled due to H5N2 virus? What are the main affected species?

|  |
| --- |
